# Microbiota-Based Intervention Alleviates High-Fat Diet Consequences Through Host-Microbe Environment Remodeling

**DOI:** 10.3390/nu17091402

**Published:** 2025-04-22

**Authors:** Lanlan Yi, Zhipeng Li, Hong Xu, Dejia Shi, Ying Huang, Hongbin Pan, Yanguang Zhao, Hongye Zhao, Minghua Yang, Hongjiang Wei, Sumei Zhao

**Affiliations:** 1 Yunnan Key Laboratory of Animal Nutrition and Feed Science, Yunnan Agricultural University, Kunming 650201, China; yilanlan0217@163.com (L.Y.); l1027994959@gmail.com (Z.L.); hying_5@163.com (Y.H.); ynsdyz@163.com (H.P.); 13808751468@163.com (M.Y.); 2 Yunnan Province Key Laboratory for Porcine Gene Editing and Xenotransplantation, Yunnan Agricultural University, Kunming 650201, China; hyzhao2000@126.com (H.Z.); hongjiangwei@126.com (H.W.); 3 School of Public Finance and Economics, Yunnan University of Finance and Economics, Kunming 650221, China; xuhong68@126.com; 4 Fuyuan Dahe Black Pig Research Institute, Qujing 655505, China; shidejia2021@163.com; 5 Shanghai Lab. Animal Research Center, Shanghai 201203, China; zyg740612@163.com; 6 College of Veterinary Medicine, Yunnan Agricultural University, Kunming 650201, China

**Keywords:** high-fat diet, obesity, intestinal, microbes

## Abstract

A high-fat diet leads to metabolic disturbances, which are important factors in the development of obesity. Gut microbial composition and diversity are altered by a high-fat diet. In general, a high-fat diet resulted in increased Firmicutes abundance and decreased alpha diversity. Bile acids (BAs) are involved in the digestion and absorption of fats in the small intestine and are also the metabolic substrates of microorganisms with bile salt hydrolase (BSH) activity. High-fat diets (HFDs) have been shown to alter gut microbiota composition and BA profiles in murine models. Similarly, probiotic supplementation reverses HFD-induced adverse effects. This review focuses on the energy composition characteristics of a high-fat diet and its effects on body weight, plasma lipid-related biochemical markers, changes in gut microbiome characteristics, and the important role of BAs. The regular mechanism by which a high-fat diet affects the intestinal microenvironment was attempted to be found.

## 1. Introduction

Obesity is a recognized global public health problem and is influenced by many factors such as age, gender, genetics, diet, geographic location, etc. [1,2,3]. A strong link between diet and obesity has been observed by changing dietary patterns [4,5]. Dietary patterns can generally be divided into three types: 1. Healthy diet, 2. Western diet, 3. Mixed diet [6]. A healthy diet is high in vegetables, fruits, and whole legumes. The Western diet is characterized by refined grains, solid fats, and snacks. A mixed diet is somewhere be-tween a healthy diet and a Western diet. It is well known that the Western diet is the main dietary pattern that contributes to obesity. Therefore, the simple summary is a high-fat diet.

The digestion and absorption of nutrients in the gut is a dynamic process in which microorganisms play an important role. The gut microbiota is an important environmental factor that affects the host’s energy acquisition and storage from the diet and can induce de novo synthesis of fat in the liver by promoting the absorption of monosaccharides in the gut [7]. There were differences in the number of microorganisms in different intestinal segments. An amount of 10^4^–10^8^ microorganisms per mL were present in the contents of the small intestine, and microbial colonization was limited due to the short residence time of the chyme. The microbial density of the large intestine can reach 10^10^ per mL, which is the intestinal segment with the highest intestinal microbial density [8]. Microbiome study is popular all over the world, and the rapid development of high-throughput sequencing technology makes it possible to observe the gut microbial environment [9]. About 90% of the microorganisms in the large intestine belong to the phyla Bacteroidetes and Firmicutes and also include Proteobacteria, Actinobacteria, Fusobacteria, and Verrucomicrobia [10].

A translational perspective is established through systematic analysis of high-fat diet-induced physiological and microbial alterations, focusing on three critical aspects: (1) the operational definition of high-fat diets, (2) identification of diet-associated gut microbiota signatures, and (3) evaluation of therapeutic potential from microbial supplementation strategies.

## 2. Nutrient Characteristics of a High-Fat Diet

### 2.1. High-Fat Diet Induces Fat Deposition

High-fat diets are characterized by low carbohydrate levels and relatively unchanged protein levels, with a fat composition of more than 30% of total dietary energy (Table 1). Body weight and adipose tissue weight increased significantly, and plasma cholesterol concentrations increased. Dietary fats are emulsified by bile salts in the small intestine, and then pancreatic juice secreted by the pancreas begins to digest the lipids. The free fatty acids produced by lipolysis are absorbed by the small intestine, resynthesize triglycerides (TGs), and enter the blood circulation in the form of chylomicrons. The TGs released in the liver are combined with apolipoproteins and transported to extrahepatic tissues for utilization [11]. At the same time, a high-fat diet also caused significant upregulation of the lipase genes adipose triglyceride lipase (*ATGL*), hormone-sensitive triglyceride lipase (*HSL*), and fatty acid synthase gene *FASN* in the liver of mice, indicating that the lipid metabolism activity of mice fed a high-fat diet was more rigorous than that of the mice fed a low-fat diet [12].

A short-term (within 2 weeks) high-fat diet has no significant effect on the body weight of mice, and the deposition of fat in the body takes a period of time. When dietary fat was increased to 50% for one week, there were no significant differences in body weight, body fat, and fat mass compared with a diet containing 30% fat [13]. Diets containing 30% vegetable oil significantly increased body weight compared with 20% [14]. A 4-week high-fat diet experiment, designed to have significantly higher cholesterol levels than the control group, then measured weekly body weights in Sprague-Dawley rats, which were nearly the same weight as the control group in the first and second weeks and began to rise slowly after the second week [15]. The feeding efficiency of C57BL/6 mice fed a high-fat diet increased, but the body mass was almost no different from the control group at 0–15 days, and the increase was less than 1 g at 15–20 days [16].

**Table 1:** Body weight, body composition, or serum biochemical markers affected by high-fat diet.

Reference	Species	Sex	Age or Weight	Groups	Energy Content	Main Fat Source of High-Fat Diet	Term	Effects
Nagai et al., 2005 [17]	human	Men	23.6 years old	Low-fat meal	70% Carbohydrates, 10% protein, and 20% fat	Butter, high-fat cream	210 min after meal	Thermoregulatory sympathetic nervous system (SNS) activity and a greater level of fat oxidation ↑ (*p* < 0.05)
				High-fat meal	20% carbohydrates, 10% protein, and 70% fat			
Meksawan et al., 2004 [13]		Men and women	Male (24.8 ± 1.0 years old) Female (22.3 ± 1.3 years old)	Regular diet	54% carbohydrates, 16% protein, and 30% fat	Not found	7 d	HDL ↑ (*p* < 0.05); no difference in body weight
				High-fat diet	31% carbohydrates, 19% protein, and 50% fat			
Rowlands and Hopkins, 2002 [18]		Men	27 ± 5 years old	High-carbohydrate diet	70% carbohydrate, 15% protein, and 15% fat	High-fat meats, eggs and dairy products, nuts and seeds, low-starch vegetable products, and oils	Three 2-week dietary treatment	During exercise: 10%–20% plasma-glucose concentration ↑ (*p* < 0.01); plasma triacylglycerol ↑ (*p* < 0.05); 2.5–2.9 fold increase in the peak fat-oxidation rate (*p* < 0.0001)
				High-fat diet	15% carbohydrate, 15% protein, and 70% fat			
Linehan et al., 2018 [15]	Sprague–Dawley rats	Male	3 weeks old	Standard diet	58.5% carbohydrates, 28.7% protein, and 12.7% fat	Not found	7~28 d	No difference in body weight
				High-fat diet	44% Carbohydrates, 16% protein, and 40% fat			
Woodie and Blythe, 2018 [19]			6 weeks old	Control diet	44.3% carbohydrate and 5.8% fat	Not found	63 d	Final body weight, body weight change, fat pad weight, food intake, and kcal consumed ↑ (*p* < 0.05); no difference in fasting blood glucose
				High-fat diet	20% carbohydrate and 60% fat			
Cheng et al., 2017 [20]	Sprague–Dawley rats	Male	3 weeks old	Control diet	70% carbohydrates, 20% protein, and 10% lipid	Corn oil, milk fat	56 d	Central obesity, systolic and diastolic hypertension, impaired fasting glucose, hypertriglyceridaemia, and elevated non-HDL cholesterol level
				High-fat diet	20% carbohydrates, 20% protein, and 60% lipid			
Huang et al., 2004 [21]	Sprague–Dawley rats	Male	7 weeks old	Standard diet	57.99% carbohydrates, 28.50% protein, and 13.49% fat	Lard	56 d	Body weight, liver weight, adipose tissue, and relative liver weight ↑ (*p* < 0.05); the plasma cholesterol concentration, a-Amylase, b-Hydroxybutyrate, and Leptin ↑
				High-fat diet	AIN-76 diet, containing 20% fat			
Kanthe et al., 2021 [22]	albino Wistar rats	Not found	180–220 g	Control diet	60% carbohydrates, 18% protein, and 20% fat	Not found	21 d	Body weight ↑ (*p* < 0.05)
				High-fat diet	50% carbohydrates, 18% protein, and 30% fat			
Patil et al., 2019 [14]				Control group	60% carbohydrate, 18% protein, and 20% fat	Vegetable oil	22 d	Final body weight ↑ (*p* < 0.05); lipid peroxidation and oxidative stress ↑
				High-fat diet	50% carbohydrate, 18% protein, and 30% fat			
Maejima et al., 2020 [23]	Wistar rats	Male	8-week-old	Normal chow diet	20.5% protein and 10.1% fat	Not found	72 d	Body weight, energy intake, visceral fat, and subcutaneous fat ↑ (*p* < 0.05); muscle ↓
				High-fat diet	20.5% protein and 56.7% fat			
He et al., 2020 [24]	Wistar rats	Male	Not found	Standard diet	55.5% carbohydrates, 33.3% protein, and 11.2% fat	Not found	70 d	No difference in TG, TC, LDL-C, and HDL-C (serum)
				High-fat diet	28.6% carbohydrates, 26.2% protein, and 45.2% fat			
Schanuel et al., 2019 [16]	C57BL/6 mice	Male	12 weeks old	Standard chow	76% carbohydrates, 14% protein, and 10% lipids	Soybean oil, lard	20 d	Body weight and average fasting blood glucose were no significant differences between groups; inflammatory and fibroblast-like cells ↑ (10 days after, *p* < 0.05)
				High-fat chow	26% carbohydrates, 14% protein, and 60% lipids			
Emelyanova et al., 2019 [25]	C57BL/6 mice	Male	6 weeks old	Standard chow	64.5% carbohydrate, 23.6% protein, and 11.9% fat	Lard	70 d	Body weight, gain of body weight ↑ (*p* < 0.05)
				High-fat diet	20% carbohydrate, 20% protein, and 60% fat			
Pang et al., 2016 [26]			8 weeks old	Normal diet	65.42% carbohydrate, 22.47% protein, and 12.11% fat	Not found	90 d	Energy efficiency ↑ (*p* < 0.01); epididymal and perirenal fat weight ↑ (*p* < 0.01); insulin and glucose concentrations ↑ (*p* < 0.05)
				High-fat diet	20% carbohydrate, 20% protein, and 60% fat			
Topal et al., 2019 [27]	Swiss albino mice	Female	8–10 weeks old	Standard chow	66% carbohydrate, 24% protein, and 10% fat	Not found	63 d	Body weight, intraperitoneal adipose tissue; adrenal gland weight ↑ (*p* < 0.01)
				High-fat diet	23% carbohydrate, 17% protein, and 60% fat			

Note: ↑ and ↓ represent higher (↑) or lower (↓) values in the HFD group compared with the standard diet group or the low-fat diet group.

Feeding a high-fat diet for more than 8 weeks resulted in obesity in mice, regardless of whether the dietary fat source was lard, vegetable oil, peanut oil, corn oil, or soybean oil. High fat led to lower food intake but higher body weight and total fat pad weight [19]. Normal obesity on a high-fat diet in childhood may develop into overweight obesity in adulthood [23].

### 2.2. Changes in Serum Lipid Indexes

TG molecules represent the major storage and transport form of fatty acids within cells and in plasma. The liver is the central organ of fatty acid metabolism. Dietary fat is mainly hydrolyzed by pancreatic lipase and then emulsified by bile acid (BA), and the resulting lipid molecules are absorbed to synthesize TG [28]. Up to 70% of dietary fat is ingested by the body, and plasma TG levels are elevated during exercise [18]. The diet containing 60% fat was fed to mice for 56 days, and the mice developed hypertriglyceridemia [20]. However, one study showed that 45.2% dietary fat did not affect the plasma levels of total cholesterol (TC), TG, low-density lipoprotein cholesterol (LDL-C), and high-density lipoprotein cholesterol (HDL-C) in male Wistar rats [24].

The type of lipids in the diet affects the levels of plasma lipid profiles. A total of 21% lard in the diet induced a significant increase in body weight gain in Wistar rats and a significant increase in TG content in the plasma lipid profile, while other parameters were not affected [29]. C57BL/6 mice were fed diets containing lard, sunflower oil, soybean oil, lard mixed with sunflower oil, and lard mixed with soybean oil for 12 weeks and found that the lard diet resulted in increased TG levels, although the vegetable oil diet did not cause obesity but will cause cholesterol metabolism disorder [30]. Oils rich in unsaturated fatty acids, such as safflower oil, sunflower oil, and rapeseed, are more effective at lowering LDL-C than foods rich in saturated fatty acids, such as butter or lard [31].

Nonalcoholic fatty liver disease (NAFLD) is caused by excessive fat deposition in liver cells caused by a high-fat diet. A mixed diet of lard and soybean oil attenuated low-fat–high-carbohydrate diet-induced NAFLD by modulating genes and BA profiles in C57BL/6 mice. A lard–soybean oil mixture alleviates NAFLD by down-regulating fatty acid binding protein 2 (*FABP2*), fatty acid synthase (*FAS*), tumor necrosis factor receptor-associated factor 2 (*TRAF2*), activator protein-1 (*AP-1*), mitochondrially encoded cytochrome b (*MT-Cytb*), interleukin 6 (*IL-6*), and interleukin 1 (*IL-1*) genes; upregulating protein kinase AMP-activated catalytic subunit alpha 2 (*AMPKα2*) and *HSL* genes and promoting the binding of BAs and BAs signaling receptor takeda G protein-coupled receptor 5 (TGR5) protein [31].

## 3. High-Fat Diet Alters Host Gut Microbiota Abundance and Diversity

### 3.1. High-Fat Diet Affects Gut Microbiome Composition

A high-fat diet will affect the composition and abundance of intestinal microbiota. The digested chyme stays in the large intestine for a long time. Therefore, more articles are studying the microbes of the large intestine and feces. Microbial composition in mouse and rat large intestines and feces was analyzed using 16S rRNA sequencing technology. When the taxonomic level is phylum, Firmicutes, Bacteroidetes, and Proteobacteria account for more than 90%. At the genus level, *Parabacteroides*, *Lachnoclostridium*, *Oscillibacter*, *Lactobacillus*, *Akkermansia*, *Bacteroides*, and *Alistipes* appeared more frequently (Table 2).

The ratio of F/B (Firmicutes/Bacteroidetes) is generally considered to be associated with obesity [51]. Some studies suggest that F/B is elevated in obese individuals, while others show no significant change in F/B [52]. This suggests that elevated F/B in obese individuals is not inevitable. If we focus solely on the changes in both phyla, the level of dietary fat content will have an impact on composition. Notably, gut microbiota composition in high-fat diet-induced obese mice exhibited distinct shifts in phylum-level dominance, with Firmicutes and Bacteroidetes showing differential responsiveness to dietary fat content. While Firmicutes prevalence was generally amplified in mice consuming elevated dietary fat levels, Bacteroidetes abundance displayed an inverse correlation pattern under equivalent conditions. However, the results in some studies demonstrated outcomes contrary to the predominant trend described above.

A high-fat diet caused increased body weight, body composition, serum TGs, and cholesterol levels in mice. The gut microenvironment is influenced by diet, and microbial composition and diversity are altered to varying degrees. Therefore, there may be a link between changes in the microbiome and body weight and serum markers. A high-fat diet can affect the composition and abundance of the gut microbiota, triggering disturbances in the gut microbiota, which may cause a range of health problems. Christensenellaceae, Porphyromonadaceae, Rikenellaceae, *Ruminococcaceae UCG 014*, and *Ruminococcaceae UCG 005* were negatively associated with obesity [50].

### 3.2. Alterations in Alpha Diversity May Be Related to Lipid Types

A considerable number of studies have shown that a high-fat diet can lead to a decrease in the Simpson, Shannon, and Chao index of gut microbes [35,37,39,41,43,47,53]. The decrease in gut microbial alpha diversity may not only be due to the effect of a high-fat diet but may also be related to interactions between lipid metabolism, inter-microbial, and microbial metabolites. Contrary to the popular belief that a high-fat diet reduces gut microbial diversity, it was elevated in a high-fat diet with lard as the main fat source compared with a low-fat diet, and linear discriminant analysis effect size (LEfSe) results indicated that the taxa features that best characterize the differences between the high-fat diet and low-fat diet groups were mainly those of the Rikenellaceae, Deferribacteraceae, Streptococcaceae, Christensenellaceae, and Peptococcaceae families [12].

High-fat diets typically use corn oil, peanut oil, soybean oil, and lard as the main fat sources. However, the fat source of the diet inducing obesity in mice was mainly lard. Different fat treatments affected the community composition of gut microbiota; for example, the abundance of Proteobacteria showed a decreasing trend in the lard, walnut, and peanut oil intervention groups, while flaxseed oil, olive oil, and canola oil showed a downward trend or increasing trend [54]. Lard has a synergistic effect with *Coriobacteriaceae_UCG-002* in the cecum of Kunming mice (half male and female), and vegetable oil has a synergistic effect with *Akkermansia*, *Roseburia*, and *Enteractinococcus*. Among them, *Coriobacteriaceae_UCG-002* showed a significant negative correlation with Glycolysis/Gluconeogenesis. *Roseburia* was most strongly associated with starch and sucrose metabolism [55].

## 4. Bile Acid—Fat Metabolism and Microbial Action

By binding to glycine (human) or taurine (rodent), BAs help limit passive reabsorption, promote micelle formation, and facilitate digestion and absorption of fats in the small intestine [56]. Bile acidolysis conjugation is carried out by bacteria with bile salt hydrolase (BSH) activity, such as *Lactobacillus*, *Bifidobacterium*, *Clostridium*, and *Bacteroidetes* with this functional BSH, resulting in a small amount of BA not being reabsorbed by the intestine back to the liver [57]. Another microbial metabolic pathway for BAs is catalyzed by bacteria with hydroxysteroid dehydrogenases found in Actinobacteria, Proteobacteria, Firmicutes, and Bacteroidetes [58].

In general, impaired gut microbial diversity affected by a high-fat diet was characterized by the highest abundance of Firmicutes, but elevated microbial diversity was characterized by the highest abundance of Bacteroidetes (Table 2). *Lactobacillus* is a member of the Firmicutes phylum. *Lactobacillus* abundance was positively correlated with the concentration of free BAs, which inhibit gut bacteria and modulate gut microflora [59]. *Parabacteroides distasonis* alleviates obesity-related metabolic dysregulation by producing succinate, which directly activates intestinal gluconeogenesis via fructose-1,6-bisphosphatase binding, paired with BA-mediated farnesoid X receptor (FXR) signaling to synergistically improve glucose and lipid homeostasis [60].

There is an interaction between microorganisms and BAs, and BAs inhibit the growth of BA-sensitive bacteria by promoting the growth of BA-metabolizing bacteria. BAs exert direct antibacterial effects through bacterial membrane damage, a mechanism demonstrable both in vitro and in vivo within the gut, and indirectly by activating ileal epithelial FXR signaling, which induces antimicrobial peptide expression in vivo [61,62]. This is one of the reasons for the altered diversity of the microbiota. In addition, a study suggests that BA metabolites produced by colonic microbes build a proinflammatory gut microenvironment that may further develop into various types of intestinal inflammation [63]. The enrichment of intestinal *Clostridia* causes an increase in the free BA content in the intestine, which stimulates the gastrointestinal tract and causes diarrhea [64].

## 5. Beneficial Effects of Probiotics on Mice Fed with High-Fat Diet

HFD has been shown to alter gut microbiota composition and BA profiles in murine models. Similarly, probiotic supplementation reverses HFD-induced adverse effects. The advent of high-throughput sequencing technologies has facilitated a surge in gut microbiome studies. Recognized as the “second genome” of animals, gut microbiota regulates a spectrum of physiological and biochemical processes. However, given the intricate regulatory networks among microbial communities, research focusing on strain-specific interventions to counteract HFD-associated dysbiosis has gained significant attention.

### 5.1. Lactobacillus

Certain strains within the *Lactobacillus* (specific strains to be subsequently described) have been identified as probiotics and have attracted considerable attention due to their ability to alleviate obesity and adipose tissue accumulation. Different strains have different effects on high-fat diet mice (Supplementary Table S1). *Lactiplantibacillus plantarum* FZU3013 reduced body weight and serum TG, TC, and LDL-c and increased the mRNA levels of liver cholesterol 7α-hydroxylase (CYP7A1) and bile salt export pump (BSEP), indicating that BA synthesis was enhanced and the excretion of BA through feces was promoted [65]. *Lactiplantibacillus plantarum* NKK20 reduced TC and TG concentrations, increased the abundance of colonic *Akkermansia* and the concentration of short-chain fatty acids (SCFAs), and regulated BA anabolism [66]. *Lactiplantibacillus plantarum* strain CNCM I-4459 reduced LDL-c concentrations and downregulated liver *FAS*, perilipin (*PLIN*), and carnitine palmitoyltransferase-I-alpha (*CPTIα*) genes [67]. *Lactiplantibacillus plantarum* FRT10 reduced body weight, fat weight, and liver TG concentration; upregulated the mRNA expression levels of liver peroxisome proliferator-activated receptor alpha (*PPARα*) and carnitine palmitoyltransferase-1 alpha (*CPT1α*); and downregulated the mRNA expression levels of liver sterol regulatory element-binding protein 1 (*SREBP-1*) and diacylglycerol acyltransferase 1 (*DGAT1*) [68]. *Lactiplantibacillus plantarum* NCHBL-004 induced glucagon-like peptide 1 (*GLP-1*) production and increased fecal SCFA levels [69]. *Lactiplantibacillus plantarum* CQPC01 inhibited the increase in adipocyte volume, increased IL-4 and IL-10 content, downregulated the expression of CCAAT/enhancer binding protein alpha (*C/EBP-α*) and peroxisome proliferator-activated receptor gamma (*PPARγ*) mRNA, and upregulated the expression of *CYP7A1*, *CPT1*, lipoprotein lipase (*LPL*), catalase (CAT), superoxide dismutase 1 (SOD1), and SOD2 [70]. *Lactiplantibacillus plantarum* SKO-001 promoted the increase in serum adiponectin, decreased the levels of leptin, insulin, TC, LDL-c, free fatty acid (FFA), and TG, and decreased the mRNA levels of *SREBP-1c* and *PPARγ* [71]. The extract of *Lactiplantibacillus plantarum* LMT1-48 reduced liver weight and TG levels and downregulated the lipogenic genes *PPARγ*, *HSL*, stearoyl-CoA desaturase-1 (*SCD-1)*, and fatty acid translocase (*FAT* or *CD36*) in the liver, leading to a reduction in body weight and fat volume [72]. *Lactiplantibacillus plantarum* CQPC03 alleviated inflammation by increasing the levels of IL-4 and IL-10 and reducing the levels of proinflammatory factors, including IL-6, IL-1β, tumor necrosis factor-alpha (TNF-α), and interferon-gamma (IFN-γ) [73]. *Lactiplantibacillus plantarum* Shinshu N-07 reduces epididymal adipose tissue weight and adipocyte area and inhibits hepatic steatosis [74]. *Lactiplantibacillus plantarum* Y44 inhibited the expression of FAS and acetyl CoA carboxylases (ACC) in the liver of obese mice, upregulated the expression of colonic tight junction proteins such as claudin-1 and occludin, reduced serum IL-8 and TNF-α levels, and increased the content of SCFA in feces [75]. *Lactiplantibacillus plantarum* HF02 inhibited pancreatic lipase activity in small intestinal contents, increased fecal TG levels, and reduced serum lipopolysaccharide (LPS), IL-1β, and TNF-α levels [76]. *Lactiplantibacillus plantarum* E2_MCCKT leads to upregulation of PPAR-α mRNA, downregulation of adipogenesis and fatty acid synthesis genes (SREBP-1c, ACC, and FAS), and downregulation of proinflammatory cytokine (IL-1Ra and TNF-α) expression [77]. *Lactiplantibacillus plantarum* KAD 8 restores metabolic health by normalizing glycemia, lipidomics, liver parameters, oxidative stress, and inflammatory parameters [78]. *Lactiplantibacillus plantarum* BXM2 reversed intestinal dysbiosis by increasing the ratio of villus height to crypt depth and the number of intestinal goblet cells and normalizing the mRNA expression of TNF-α and IL-6 [79]. *Lactiplantibacillus plantarum* DSR330 reduced the expression of SREBP-1c, ACC1, FAS, 1-aminocyclopropane-1-carboxylicacid oxidase (ACO), PPARα, and CPT-1 in hepatocytes [80]. *Lactiplantibacillus plantarum* A29 downregulated the expression of lipogenic genes (PPAR-γ, C/EBP-α, and C/EBP-β) in adipocytes and alleviated the development of obesity by increasing phosphorylation and activation of p38 mitogen-activated protein kinase (MAPK), p44/42, and AMPK-α [81]. *Lactiplantibacillus plantarum* dfa1 reduced inflammatory cytokines in blood and colon tissue and decreased the relative abundance of Proteobacteria [82]. *Lactiplantibacillus plantarum* strain Ln4 reduced body weight and epididymal fat mass and decreased the protein levels of C-reactive protein (CRP), insulin-like growth factor binding protein-3 (IGFBP-3), and monocyte chemoattractant protein-1 (MCP-1) in white adipose tissue [83]. *Lactiplantibacillus plantarum* DSM20174 improved glucose and lipid homeostasis and reduced white adipose inflammation [84]. *Lactiplantibacillus plantarum* KC28 significantly upregulated PPAR-gamma co-activator-1 alpha (PGC1-α) and CPT1-α in the liver and downregulated ACOX-1, PPAR-γ, and FAS expression in mesenteric adipose tissue [85].

*Lacticaseibacillus paracasei* S0940 and *Streptococcus thermophilus* ldbm1 reduced serum and liver TC and TG levels in high-fat-fed mice [86]. *Bifidobacterium longum* BORI reduced the body weight of mice, *Lactobacillus acidophilus* AD031 and *Bifidobacterium bifidum* BGN4 reduced the TG level in the liver of mice, while *Bifidobacterium longum* BORI reduced the TC level in the liver [87]. *Lacticaseibacillus paracasei* 24 reduced body weight and fat deposition, decreased the ratio of Firmicutes/Bacteroidetes, and increased the abundance of *Akkermansia* [88]. *Lacticaseibacillus paracasei* K56 reduced the expression of FAS and PPAR-γ in the liver [89]. *Lacticaseibacillus paracasei* N1115 reduced visceral fat, liver weight, serum insulin, and leptin levels; altered intestinal microbiota; and increased SCFA content [90]. *Lacticaseibacillus paracasei* X-1, *Lacticaseibacillus paracasei* X-17, and *Limosilactobacillus fermentum* BM-325 inhibited the growth of adipocyte volume and stabilized fasting blood glucose [91]. *Lacticaseibacillus paracasei* BEPC22 and *Lactiplantibacillus plantarum* BELP53 reduced white adipose tissue volume and adipocyte size, reduced the expression of PPARγ in the liver, and increased the expression of PPARα in white adipose tissue [92]. *Lacticaseibacillus paracasei* FZU103 reduced epididymal adipocyte hypertrophy, promoted fecal excretion of BAs, and increased the relative abundance of *Ruminococcus*, *Alistipes*, *Pseudoflavonifractor* and *Helicobacter* [93]. *Lacticaseibacillus paracasei* AO356 alters the relative abundance of microorganisms involved in lipid metabolism pathways and obesity-related markers, such as *Lactobacillus*, *Bacteroides*, and *Oscillospira* [94]. *Lactobacillus casei* CRL 431 exerts beneficial effects by reducing the proinflammatory cytokines IL-6, IL-17, and TNF-α [95].

*Lactobacillus sakei* MJM60958 reduced the expression of FAS, ACC, and SREBP-1 in the liver; upregulated the expression of PPARα and CPT1A; and affected the regulation of intestinal flora by increasing the production of acetate [96]. *Latilactobacillus sakei* QC9 increased the abundance of butyrate-producing bacteria and the content of SCFA to mediate the microbiota-gut-liver axis, affecting the phosphatidylinositol 3-kinase (PI3K)/Akt signaling pathway in the liver and alleviating the development of T2DM [97]. *Latilactobacillus sakei* WIKIM31 reduced body weight gain, epididymal fat mass, TG, and TC levels; significantly decreased the expression of lipogenesis-related genes in epididymal adipose tissue and liver; and promoted the production of intestinal short-chain fatty acids (such as butyrate and propionate) [98]. *Lactobacillus sakei* OK67 reduced LPS levels in blood and colon contents, colonic TNF-α and IL-1β expression, and nuclear factor-kappaB (NF-κB) activation; increased IL-10 and tight junction protein expression; and downregulated PPARγ, FAS, and TNF-α expression in adipose tissue [99,100]. *Lactobacillus sakei* ADM14 reduced body weight gain and blood glucose levels, decreased the expression of lipid-related genes in the epididymal fat pad, decreased the ratio of Firmicutes to Bacteroidetes, and increased the relative abundance of *Bacteroides faecichinchillae* and *Alistipes* [40]. *Lactobacillus sakei* CJLS03 reduced the average size of adipocytes, decreased the gene expression of SREBP-1c, FAS, and SCD1 in epididymal adipose tissue, and increased the levels of SCFA in serum and feces [101].

*Lactobacillus acidophilus* reduces body weight, fat mass, inflammation, and insulin resistance and inhibits the toll-like receptor 4 (TLR4)/NF-κB signaling pathway [102]. *Lactobacillus acidophilus* NX2-6 activated the insulin signaling pathway; promoted glucose uptake, glycolysis, and intestinal gluconeogenesis; inhibited hepatic gluconeogenesis; effectively lowered blood glucose levels and improved glucose tolerance; and improved liver energy metabolism through the fibroblast growth factor 21 (FGF21)/AMPKα/PGC-1α/ nuclear respiratory factor 1 (NRF1) pathway [103]. *Lactobacillus acidophilus* NS1 increased the expression of SREBP2 and low-density lipoprotein receptor (LDLR) in the liver [104]. *Lactobacillus acidophilus* LA5 reduced obesity, intestinal permeability defect, endotoxemia, and serum cytokines in mice and increased the relative abundance of *Akkermansia muciniphila* [105]. *Lactobacillus acidophilus* GOLDGUT-LA100 has high BSH activity, good gastric acid and bile salt tolerance, and alleviates the pathophysiological symptoms of high-fat diet-induced obese mice [106].

*Lactobacillus fermentum* CECT5716 increased the relative abundance of *Akkermansia* sp. and the proportion of Bacteroidetes [107]. *Limosilactobacillus fermentum* HNU312 reduced body weight, serum TG, TC, and LDL-c levels, significantly reduced fat accumulation in the liver and adipose tissue, and increased SCFA production [108]. *Limosilactobacillus fermentum* MG4231 reduced the expression of PPARγ, C/EBPα, FAS, PLIN2, and LPL in epididymal tissue and reduced SREBP1-c and FAS in liver tissue [109]. *Limosilactobacillus fermentum* MG4294 and *Lactiplantibacillus plantarum* MG5289 reduced the levels of proinflammatory cytokines TNF-α, IL-1β, and IL-6 in intestinal tissues [110]. *Lactobacillus fermentum* LM1016 improved glucose clearance and fatty liver and reduced inflammation in gonadal white adipose tissue in mice fed a high-fat diet [111]. *Lactobacillus fermentum* CKCC1858, *Lactobacillus fermentum* CKCC1369, *Lactiplantibacillus plantarum* CKCC1312, and *Lactobacillus gasseri* CKCC1913 alleviated liver and pancreatic damage, reduced blood lipids and the secretion of proinflammatory cytokines, increased liver antioxidant enzymes, and improved hyperlipidemia, inflammation, and oxidative stress [112].

*Lactobacillus rhamnosus* reduced serum IL-6 levels [113]. *Lactobacillus rhamnosus* TR08 promoted the increase in the relative abundance of Bifidobacteria and Bacteroidetes, thereby reshaping the intestinal flora, reducing the abundance of pathogenic bacteria *Enterococci*, and increasing the content of SCFAs [114]. *Lactobacillus rhamnosus* strain LRH05 modulated white adipose tissue monoacylglycerol O-acyltransferase 1 (*MOGAT1*), insulin-like growth factor-1 (*IGF-1*), *MCP-1*, and *F4/80* mRNA expression and increased butyrate- and propionate-producing bacteria (*Lachnoclostridium*, *Romboutsia*, and *Fusobacterium*) [115]. *Lactobacillus rhamnosus* LA68 significantly reduced TC and HDL levels, while *Lactiplantibacillus plantarum* WCFS1 was more effective in reducing TG and LDL levels [116]. *Lactobacillus rhamnosus* GG restored exogenous leptin responsiveness, increased the ratio of villus height to crypt depth, and reduced the proportion of Proteobacteria in the fecal microbiota [117]. *Lactobacillus rhamnosus* LRa05 reduced body weight, blood lipid levels, and lipid accumulation in hepatocytes and epididymal adipose tissue; reduced the abundance of the pathogen-promoting bacterium *Streptococcus*; and inhibited blood and liver glucose content [118]. *Lactobacillus rhamnosus* GR-1 reduced the development of oxidative stress and chronic inflammation in a dose-dependent manner [119].

*Lactobacillus gasseri* SBT2055 reduced the number of macrophages in adipose tissue, the ratio of M1 macrophages to total macrophages was significantly reduced, and the expression of C-C motif ligand 2 (CCL2), C-C chemokine receptor 2 (CCR2), and leptin (LEP) was downregulated [120]. *Lactobacillus paragasseri* SBT2055 increased small intestinal lipid excretion into feces by reducing the mRNA levels of *FABP1*, *FABP2*, fatty-acid transport protein 4 (*FATP4*), *CD36*, and apolipoprotein B48 (*APOB48*) [121].

*Lactobacillus johnsonni* 3121 and *Lactobacillus rhamnosus* 86 downregulated the expression of genes related to adipogenesis and normalized the obesity-associated intestinal microbiota [122]. *Lactobacillus johnsonii* JNU3402 reduced the expression of hepatic SREBP-1c, FAS, and ACC; inhibited SREBP-1c transcriptional activity by enhancing protein kinase A (PKA)-mediated phosphorylation; and reduced the expression of its lipogenic target genes in alpha mouse liver 12 (AML12) and HepG2 cells, thereby attenuating hepatic lipid accumulation [123].

*Lactobacillus curvatus* HY7601 and *Lactiplantibacillus plantarum* KY1032 upregulate the expression of cholesterol transport genes in the liver and jejunum, including liver X receptors alpha (LXRα), ATP-binding cassette transporters G (ABCG) 5 and ABCG8, and CYP7A1 [124]. *Lactobacillus curvatus* HY7601 and *Lactiplantibacillus plantarum* KY1032 upregulate the expression of fatty acid oxidation-related genes (PGC1α, CPT1, CPT2, and ACOX1) in the liver and reduce the expression of proinflammatory genes (TNFα, IL6, IL1β, and MCP1) in adipose tissue [125].

*Lactobacillus reuteri* FN04 reduces hepatic FAS overexpression, increases SREBP1c expression, improves intestinal epithelial barrier function, and induces the intestinal microbiota to produce SCFAs [126]. *Limosilactobacillus reuteri* BIO7251 reduced subcutaneous adipose tissue mass, glucose absorption, and food intake [127]. *Lactobacillus rhamnosus* FJSYC4-1 and *Lactobacillus reuteri* FGSZY33L6 alleviated weight gain, blood sugar, and lipid disorders; regulated intestinal flora; and produced SCFA [128].

*Lactobacillus amylovorus* KU4 increased the expression of uncoupling protein 1 (UCP1), PPARγ, and PGC-1α in subcutaneous inguinal white adipose tissue, reduced receptor-interacting protein 140 (RIP140) expression, and released RIP140 to stimulate UCP1 expression, thereby increasing the interaction between PPARγ and PGC-1α, thereby promoting the browning of white adipocytes [129]. *Ligilactobacillus Salivarius* LCK11 inhibited food intake by significantly increasing the transcription and translation levels of peptide tyrosine tyrosine (PYY) and, ultimately, the serum PYY level, which is attributed to the activation of the TLR2/NF-κB signaling pathway in enteroendocrine L cells by the peptidoglycan of LCK11 [130]. *Lactobacillus kefiri* DH5 upregulated the expression of PPAR-α, FABP4 and CPT1 in epididymal adipose tissue, stimulated fatty acid oxidation, and reduced obesity [131]. *Lactobacillus pentosus* S-PT84 improved intestinal integrity by maintaining tight junction protein expression to inhibit LPS from entering the blood and reduced the secretion of TNF-α and MCP-1 to inhibit systemic inflammatory response [132]. *Lactobacillus delbrueckii* subsp. *lactis* CKDB001 reduced liver TG and TC levels without significantly affecting the expression of genes related to lipid metabolism [133]. *Lactobacillus coryniformis* supsp. *torquens* T3 inhibits liver inflammation and oxidative stress damage by regulating the LPS inflammatory pathway in the liver, enhances the mechanical function of the intestinal barrier, and increases the content of short-chain fatty acids [134].

### 5.2. Bifidobacterium (Supplementary Table S2)

*Bifidobacterium longum* subsp. *infantis* YB0411 reduced body weight and fat weight [135]. *Bifidobacterium longum* subsp. *infantis* FB3-14 reduced the Firmicutes/Bacteroidetes ratio and increased the abundance of *Akkermansia muciniphila*, unclassified_Muribaculaceae, *Lachnospiraceae_NK4A136_group*, and *Bifidobacterim* [136]. *Lactiplantibacillus plantarum* LC27 and *Bifidobacterium longum* LC67 increased the expression of claudin-1 and occludin in the colon and reduced the level of Firmicutes and Proteobacteria and fecal LPS [137]. *Bifidobacterium longum* subsp. *longum* BL21 reduced serum TC, TG, and LDL-c levels, improved fat vacuolization in hepatocytes and epididymal fat accumulation, and reduced the Firmicutes/Bacteroidetes ratio [138].

*Bifidobacterium adolescentis* IM38 increased colonic IL-10 and tight junction protein expression, downregulated NF-κB activation and TNF expression, and reduced blood and colonic content LPS levels, as well as the ratio of Proteobacteria to Bacteroidetes [139]. *Bifidobacterium adolescentis* (BA3, BA5, Z25) and *Lactobacillus rhamnosus* (LGG, L7-1, L10-1) increased the concentration of SCFA in the intestine of mice, among which *Lactobacillus rhamnosus* LGG regulated energy metabolism and lipid metabolism, and *Lactobacillus rhamnosus* L10-1 reduced liver inflammation [140].

*Bifidobacterium animalis* subsp. *lactis* lkm512 improved hepatic lipid accumulation and intestinal barrier function [141]. *Bifidobacterium animalis* subsp. *lactis* MN-Gup significantly reduced fasting blood glucose levels, increased SCFA levels, increased the relative abundance of *Bifidobacterium*, and reduced the relative abundance of *Escherichia-Shigella* and *Staphylococcus* [142].

*Bifidobacterium lactis* IDCC 4301 reduced body weight and adipose tissue weight, increased blood lipid levels, and downregulated mRNA expression of adipogenesis-related genes [143]. *Bifidobacterium breve* strain B-3 dose-dependently inhibited body weight and epididymal fat accumulation, increased serum TC, fasting blood glucose, and insulin levels, and significantly increased intestinal *bifidobacterium* counts [144]. *Bifidobacterium* CECT 7765 reduced serum levels of leptin, IL-6, and monocyte chemotactic protein-1 while increasing IL-4 levels [145]. *Bifidobacterium bifidum* DS0908 reduced body weight and epididymal fat accumulation and serum TG, LDL-c, and TC levels [146].

### 5.3. Other Probiotics (Supplementary Table S3)

*Bacillus coagulans* BC69 reduced body weight and increased acetate and butyrate concentrations in feces [147]. A probiotic mixture consisting of five different *Bacillus* species (*sonorensis* JJY12-3, *paralicheniformis* JJY12-8, *sonorensis* JJY13-1, *sonorensis* JJY 13-3, and *sonorensis* JJY 13-8) increased the hepatic expression of lipid oxidation genes, downregulated the expression of genes for lipid uptake and lipogenesis, and reduced lipid accumulation in subcutaneous and mesenteric adipose tissue [148]. *Bacillus amyloliquefaciens* SC06 improved the antioxidant capacity of mice through the Nrf2/Keap1 signaling pathway and reduced the ratio of Firmicutes/Bacteroidetes [149]. *Bacillus coagulans* T4 inhibits the accumulation of macrophages in white adipose tissue, converts M1 macrophages into M2 macrophages, reduces TLR4 gene mRNA expression, increases the number of *Lactobacillus* and *Faecalibacterium*, and increases propionate and acetate levels [150]. *Bacillus licheniformis* reduces body weight, serum and liver TG, and epididymal fat weight; reduces liver fat deposition; and significantly changes the colonic bacterial community of obese mice [151].

*Bacteroides vulgatus* leads to reduced 5-hydroxytryptamine (5-HT) synthesis in jejunal enterochromaffin cells and reduced chylomicron uptake in the jejunal mesentery after HFD in Tph1ΔIEC, thereby alleviating HFD-induced obesity and metabolic dysfunction [152]. *Bacteroides thetaiotaomicron* increases the proportion of polyunsaturated fatty acids in the liver and prevents hepatic steatohepatitis and liver damage [153]. *Bacteroides ovatus* reduces serum LPS, CD163, IL-1β, and TNF-α levels and downregulates genes for de novo lipogenesis in the liver (SREBF1, ACC, SCD1, and FAS), accompanied by upregulation of genes related to fatty acid oxidation (PPARα) [154].

*Pediococcus pentosaceus* PP04 reduces serum TC, TG, LDL-C, FFA, leptin, LPS, and TNF-α levels; downregulates liver SREBP-1c, FAS, and SCD1 to inhibit lipogenesis; and significantly increases the expression of tight junction proteins such as occludin, claudin-1 and zonula occludens-1 (ZO-1) to improve the abnormal increase in intestinal permeability, thereby reducing liver LPS concentration and alleviating intestinal inflammation caused by a high-fat diet through the NF-κB/Nrf2 signaling pathway [155,156]. *Clostridium cochlearium* reduces body weight, fat mass, fasting blood glucose, and SCFA levels [157]. *Clostridium tyrobutyricum* reduces liver PPARγ expression, upregulates AMPK, PPARα, ATGL, and HSL expression, reduces the expression of TNF-α, IL-6, and IL-1β in the colon, and upregulates the expression of tight junction proteins [158].

*Enterococcus faecium* SF68 improves intestinal barrier integrity and function in obese mice by increasing the expression of tight junction proteins and intestinal butyrate transporter [159]. *Blautia producta* can inhibit cellular lipid accumulation and improve hyperlipidemia [160]. *Leuconostoc mesenteroides* subsp. *mesenteroides* SD23 reduces the height of intestinal villi, reduces the expression of TNF-α in the liver, and increases the expression of IL-10 [161]. *Roseburia hominis* inhibits the expansion of white adipose tissue in mice fed a high-fat diet, which is partly attributed to the production of nicotinamide riboside and the upregulation of the Sirtuin1/mTOR signaling pathway [162]. *Coprococcus* can effectively reverse HFD-induced hepatic lipid accumulation, inflammation, and fibrosis in mice [163]. *Akkermansia muciniphila* alleviated high-fat diet-induced weight gain, hepatic steatosis, and liver damage; decreased *Alistipes*, *Lactobacilli*, *Tyzzerella*, *Butyricimonas*, and *Blautia*; and increased *Clostridium*, *Osclibacter*, *Allobaculum*, *Anaeroplasma*, and *Rikenella*. *Akkermansia muciniphila* regulated the intestinal FXR-FGF15 axis and remodeled BA construction, reducing secondary BAs in the cecum and liver, including deoxycholic acid (DCA) and lithocholic acid (LCA) [164].

## 6. Summary

A high-fat diet is the main cause of obesity. Containing more than 30% dietary fat can be called a high-fat diet. Short-term high-fat diets of less than 2 weeks resulted in almost no weight gain, while more than 8 weeks resulted in a significant increase in fat deposition. Dietary fat is digested and absorbed into the blood in the small intestine, and when it reaches a certain amount, it will cause an increase in TC, TG, LDL-C, and HDL-C in plasma, which is related to many metabolic diseases.

High-fat diets lead to altered gut microbiome profiles. Firmicutes and Bacteroidetes usually account for more than 90%. The F/B ratio in obese mice is generally thought to be elevated, but there are exceptions. Gut microbiota composition in high-fat diet-induced obese mice exhibited distinct shifts in phylum-level dominance, with Firmicutes and Bacteroidetes showing differential responsiveness to dietary fat content. A high-fat diet leads to a decrease in the alpha diversity of gut microbes, and different lipid types may influence changes in microbial abundance. Family-level microorganisms such as Rikenellaceae, Deferribacteraceae, Streptococcaceae, Christensenellaceae, and Peptococcaceae may be important biomarkers that differentiate high-fat diets from normal diets.

BAs are key to the emulsification of fats in the small intestine. Small amounts of BAs are conjugated by BSH-active microorganisms in the gut, including *Lactobacillus*, *Bifidobacterium*, *Clostridium*, and *Bacteroides*, thereby preventing their entry into the enterohepatic circulation. Decreased gut microbial alpha diversity related to the levels of BAs. Adequate BA levels are beneficial for maintaining gut health and weight management. Supplementing probiotics can reverse the negative effects of obesity caused by a high-fat diet in mice, including weight gain, increased serum TG, TC, and LDL contents, increased adipocyte area, increased gene expression of lipogenic genes in the liver and adipose tissue, and a damaged intestinal barrier.

**Figure 1:**
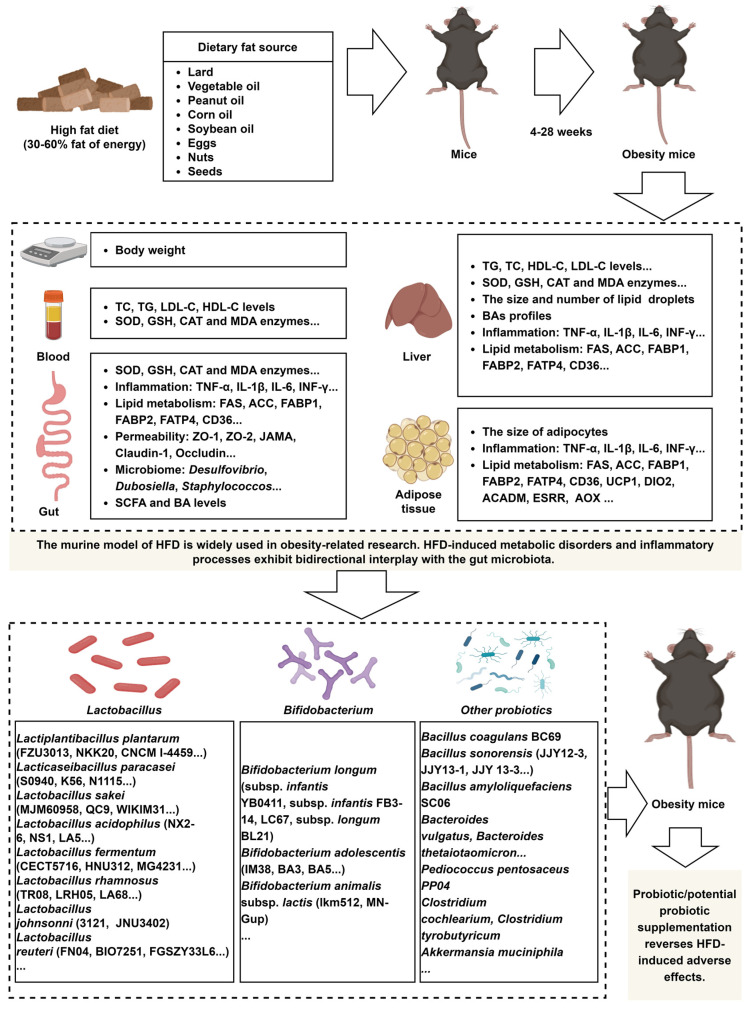
A HFD induces dysregulation of lipid metabolism and immune responses in mice, and probiotic supplementation effectively reverses these detrimental effects. The image material in Figure 1 comes from www.biorender.com.

The murine model of HFD is widely used in obesity-related research. HFD-induced metabolic disorders and inflammatory processes exhibit bidirectional interplay with the gut microbiota (Figure 1). The advancement of high-throughput technologies has led to a growing number of studies investigating microbial strains that mitigate the adverse effects of HFD, although most of these strains belong to the *Lactobacillus*. However, research on strains from genera identified by 16S rRNA sequencing and metagenomic analyses as taxa potentially linked to probiotic properties, such as *Bifidobacterium*, *Akkermansia*, *Prevotella*, and *Osclibacter*, remains limited, as these techniques primarily reveal taxonomic associations rather than direct evidence of probiotic functions. Future studies should prioritize mechanistic investigations of strains within these genera to evaluate their probiotic potential.

## Figures and Tables

**Table 2:** Gut microbial composition under high-fat diet treatment.

Reference	Species	Sex	Age or Weight	Control Group (CG) Diet	High-Fat Diet	Terms	Sample	Dominant Flora of HF	Compared with the CG	Diversity (HF vs. CG)
Song et al., 2021 [32]	C57BL/6 mice	Male	4 weeks old	70% carbohydrate, 10% fat, 20% protein	35% carbohydrate, 45% fat, 20% protein	14 weeks	Feces	Phylum: about 66% Firmicutes, 27% Bacteroidetes, 5% Proteobacteria, and 1% Tenericutes Genus: *Parabacteroides*, *Clostridiales*, *Lactobacillus*, *Akkermansia*, *Bacteroides*, and *Alistipes*	Increase: Firmicutes, Proteobacteria, Tenericutes, and *Lactobacillus* Reduce: Bacteroidetes, Parabacteroides, *Akkermansia*, *Bacteroides*, and *Alistipes*	Alpha: Not found Beta: the respective aggregation areas do not overlap
Han et al., 2021 [33]	C57BL/6 mice	Male	19–22 g	55.9% carbohydrate, 5.2% fat, 18% protein	41% carbohydrate, 30% fat, 13% protein	14 weeks	Feces	Phylum: about 40% Bacteroidetes, 29% Firmicutes, and 24% Proteobacteria Genus: Not found	Increase: Firmicutes and Proteobacteria Reduce: Bacteroidetes	Alpha: Not found Beta: the respective aggregation areas do not overlap
Lu et al., 2021 [34]	C57BL/6 mice	Male	7 weeks old	70% carbohydrate, 10% fat, 20% protein	20% carbohydrate, 60% fat, 20% protein	12 weeks	Feces	Phylum: about 55% Firmicutes, 40% Bacteroidetes, and 2% Proteobacteria Genus: *Alistipes*, *Blautia*, *Oscillibacter*, *Rikenella*, *Ruminiclostridium*, *Ruminococcaceae_UCG-014*, *Lachnoclostridium*, *Lactococcus*, *Streptococcus*	Increase: Firmicutes, *Oscillibacter* Reduce: Bacteroidetes, Proteobacteria	Alpha: Not found Beta: the respective aggregation areas do not overlap
Wu et al., 2021 [35]	C57BL/6 mice	Male	20 ± 2 g	73.5% corn, 20% wheat bran, 5% fish meal, 1% farina, and 0.5% salt	20.8% carbohydrate, 60.9% fat, 18.3% protein, and 228 mg/kg cholesterol	16 weeks	Feces	Phylum: Firmicutes, Bacteroidetes, TM7, Tenericutes, and Actinobacteria Genus: *Anaerostipes*, *Coprococcus*, *Blautia*, *Oscillospira*, *Ruminococcus*, *Allobaculum*, *Clostridium*, *Lactobacillus*, *Parabacteroides*, *Paraprevotella*, *Prevotella*, *Odoribacter*, *Butyricimonas*, *AF12*, *Bacteroides*, *Desulfovibrio*, *Bilophila*, and *Bifidobacterium*	Increase: *Anaerostipes*, *Coprococcus*, *Blautia*, *Oscillospira*, *Ruminococcus*, *Allobaculum*, *Clostridium*, *Odoribacter*, *Butyricimonas*, *AF12*, *Bacteroides*, *Desulfovibrio*, and *Bilophila* Reduce: *Lactobacillus*, *Parabacteroides*, *Paraprevotella*, *Prevotella*, and *Bifidobacterium*	Alpha: Chao1, Observed species, Shannon ↓ Beta: CG and HF groups formed two distinct clusters
Islam et al., 2021 [36]	C57BL/6 mice	Male	5 weeks old	Not found	35% carbohydrate, 45% fat, 20% protein	14 weeks	Cecal contents	Phylum: about 57% Firmicutes, 30% Bacteroidetes, and 12% Verrucomicrobia Family: Lachnospiraceae, Muribaculaceae, Ruminococcaceae, Akkermansiaceae, Erysipelotrichaceae, Bacteroidaceae, Clostridiaceae 1, Peptostreptococcaceae, and Burkholderiaceae	Not found	Not found
Peng et al., 2020 [37]	C57BL/6 mice	Male	6 weeks old	70% carbohydrate, 10% fat, 20% protein	35% carbohydrate, 45% fat, 20% protein	12 weeks	Feces	Phylum: about 54% Bacteroidetes, and 42% Firmicutes Genus: *Ruminococcaceae_UCG_014*, *Eisenbergiella*, *Faecalibaculum*, *Prevotellaceae_UCG_001*, *Alloprevotella*, *Akkermansia*, and *Ruminococcus_2*	Increase: Bacteroidetes, *Ruminococcaceae_UCG_014*, *Eisenbergiella*, *Faecalibaculum*, *Prevotellaceae_UCG_001*, *Alloprevotella*, *Akkermansia*, and *Ruminococcus_2* Reduce: Firmicutes	Alpha: Sobs ↑(*p* < 0.01), Shannon ↑(*p* > 0.05) Beta: the respective aggregation areas do not overlap
Peng et al., 2020 [37]	C57BL/6 mice	Female	6 weeks old	70% carbohydrate, 10% fat, 20% protein	35% carbohydrate, 45% fat, 20% protein	12 weeks	Feces	Phylum: about 62% Firmicutes, 27% Bacteroidetes, and 8% Proteobacteria Genus: *Escherichia_Shigella*, *Blautia*, *Parabacteroides*, *Erysipetatoclostridium*, *Anaerotruncus*, *Ruminiclostridium_9*, *Lachnoclostridium*, *Ruminococcaceae_UCG_004*, *Streptococcus*, *Lactococcus*, and *Acinetobacter*	Increase: Proteobacteria, *Escherichia_Shigella*, *Blautia*, *Parabacteroides*, *Erysipetatoclostridium*, *Anaerotruncus*, *Ruminiclostridium_9*, *Lachnoclostridium*, *Ruminococcaceae_UCG_004*, *Streptococcus*, *Lactococcus*, and *Acinetobacter* Reduce: Firmicutes and Bacteroidetes	Alpha: Sobs ↓ (*p* < 0.01), Shannon ↓ (*p* < 0.05) Beta: the respective aggregation areas do not overlap
Wang et al., 2020 [38]	C57BL/6 mice	Male	6 weeks old	Containing 10% fat by energy	Containing 60% fat by energy	16 weeks	Feces	Phylum: about 70% Firmicutes, 17% Bacteroidetes, 14% Proteobacteria Genus: *Allobaculum*, *Bacteroides*, *Lachnospiraceae_NK4A136_group*, *Desullovibrio*, *Ruminiclostridium_9*, *Alistipes*, *Coriobacteriaceae_UCG-002*, *Lactobacillus*, *Helicobacter*, and *Alloprevotella*	Increase: Proteobacteria, *Bacteroides*, *Lachnospiraceae_NK4A136_group*, *Desullovibrio*, *Ruminiclostridium_9*, *Alistipes*, *Coriobacteriaceae_UCG-002*, *Lactobacillus*, and *Helicobacter* Reduce: Firmicutes, Bacteroidetes, and *Allobaculum*	Alpha: Not found Beta: the respective aggregation areas do not overlap
Xu et al., 2020 [39]	C57BL/6 mice	Male	4 weeks old	Normal chow diet	20% carbohydrate, 60% fat, 20% protein	9 weeks	Feces	Phylum: about 57% Firmicutes, 36% Bacteroidetes, 5% Proteobacteria, and 1% Verrucomicrobia Genus: *Allobaculum*, *Bacteroides*, *Lactococcus*, *Parabacteroides*, and *Odoribacter*	Increase: Firmicutes, Proteobacteria, Verrucomicrobia, and *Odoribacter* Reduce: Bacteroidetes, *Bacteroides*, and *Parabacteroides*	Alpha: Observed species, Chao, ACE, and Simpson ↓ Beta: the respective aggregation areas do not overlap
Won et al., 2020 [40]	C57BL/6 mice	Male	5 weeks old	Containing 10% fat by energy	Containing 60% fat by energy	10 weeks	Cecal contents	Phylum: about 44% Firmicutes, 28% Verrucomicrobia, 22% Proteobacteria, and 3% Bacteroidetes Family: Lachnospiraceae, Ruminococcaceae, Desulfovlbrionaceae, Bacteridaceae, Helicobacteraceae, and Muribaculaceae	Increase: Firmicutes, Verrucomicrobia, Lachnospiraceae, and Desulfovlbrionaceae Reduce: Bacteroidetes, Proteobacteria, Ruminococcaceae, Helicobacteraceae, and Muribaculaceae	Alpha: No significant change in the alpha diversity index Beta: the respective aggregation areas do not overlap
Jing et al., 2022 [11]	C57BL/6 mice	Male	7 weeks old	70% carbohydrate, 10% fat, 20% protein	35% carbohydrate, 45% fat, 20% protein	8 weeks	Cecal contents	Phylum: Firmicutes, Proteobacteria, Actinobacteria, Bacteroidetes Genus: *Aerococcus*, *Staphylococcus*, *Bacteroides*, *Adlercreutzia*, *Alistipes*, *Akkermansia*, *Parabacteroides*, *Turicibacter*, *Lachnospiraceae_NK4A136_group*, and *norank_f_Lachnospiraceae*	Increase: Proteobacteria, Actinobacteria, *Turicibacter*, and *Lachnospiraceae_NK4A136_group* Reduce: Firmicutes, Bacteroidetes, *Aerococcus*, *Adlercreutzia*, *Alistipes*, *Akkermansia*, *Parabacteroides*, and *norank_f_Lachnospiraceae*	Alpha: Chao, Shannon ↓ Beta: the respective aggregation areas do not overlap
Van et al., 2020 [41]	C57BL/6 mice	Male	9 weeks old	70% carbohydrate, 10% fat, 20% protein	Containing 60% fat by energy	8 weeks	Cecal contents	Phylum: Firmicutes, Bacteroidetes, Proteobacteria, and Verrucomicrobia Genus: *Akkermansia*, *Allobaculum*, *Ruminococcus*, *Oscillospira*, *Odoribacter*, *Parabacteroides*, and *Bacteroides*	Increase: *Ruminococcus*, *Oscillospira*, and *Odoribacter* Reduce: *Allobaculum*, *Parabacteroides*, and *Bacteroides*	Alpha: Shannon, Simpson, Chao 1 ↓ Beta: the respective aggregation areas do not overlap
Gu et al., 2019 [42]	C57BL/6 mice	Male	6 weeks old	48.2% cornstarch, 16.1% maltodextrin, 6.4% sucrose, 2.4% soybean oil, 1.6% lard	22.5% maltodextrin, 8.9% sucrose, 3.3% soybean oil, 30.1% lard	8 weeks	Cecal contents	Phylum: about 52% Firmicutes, 42% Bacteroidetes, and 5% Proteobacteria Genus: *Butyricimonas*, *Butyricicoccus*, *Rikenella*, *Anaerotruncus*, *Xylanibacter*, and *Parasutterella*	Increase: Bacteroidetes, Proteobacteria, *Butyricimonas*, *Rikenella*, *Xylanibacter*, *Butyricicoccus*, *Anaerotruncus*, and *Parasutterella* Reduce: Firmicules, *Oscillibacter*, *Lachnospiracea_incertae_sedis*, *Flavonifractor*, *Helicobacter*, *Bacteoides*, and *Pseudoflavonifractor*	Alpha: no significant difference in Shannon and Chao1 Beta: the respective aggregation areas do not overlap
Liu et al., 2019 [43]	C57BL/6 mice	Male	7 weeks old	Containing 9.4% fat by energy	Containing 40% fat by energy	28 weeks	Feces	Phylum: 53% Bacteroidetes, 39% Firmicutes, 7% Proteobacteria, and 1% Verrucomicrobia Family: Bacteroidales_S24-7_group, Lachnospiraceae, Ruminococcaceae, Rikenellaceae, Bacteroiddaceae, Erysipelotrichaceae, Porphyromonadaceae, Lactobacillaceae, Helicobacteraceae, Desulfovibrionaceae, Verrucomicrobiaceae, and Peptostreptococcaceae	Increase: Bacteroidetes, Proteobacteria, Bacteroidales_S24-7_group, Ruminococcaceae, Rikenellaceae, Helicobacteraceae, Desulfovibrionaceae, and Peptostreptococcaceae Reduce: Firmicutes, Verrucomicrobia, Lachnospiraceae, Erysipelotrichaceae, Lactobacillaceae, and Verrucomicrobiaceae	Alpha: Sobs, Shannon ↓ Beta: the respective aggregation areas do not overlap
Cao et al., 2020 [44]	C57BL/6 mice	Male	4 weeks old	All mice were fed a high-fat diet, and fat mice were compared with lean mice	7.2% cornstarch, 9.9% maltodextrin, 17.0% sucrose, 5.5% soybean oil, 39.4% lard	16 weeks	Colon contents	Phylum: about 56% Bacteroidetes, 35% Firmicutes and 2% Actinobacteria Genus: about 12% *Alistipes*, 10% *Bacteroides*, 6% *Oscillibacter*, 5% *Ruminiclostridium*, 3% *Lachnospiraceae*, 2% *Lactobacillus*, and 2% *Faecalibaculum*	Increase: Bacteroidetes, *Alistipes*, *Oscillibacter*, *Ruminiclostridium*, *Odoribacter*, and *Alloprevotella* Reduce: Firmicutes, *Faecalibaculum*, *Lactobacillus*, *Bacteroides*, *Lachnospiraceae*, and *Akkermansia*	Alpha: Simpson ↑ (*p* < 0.05); no significant difference in Sobs, Shannon, Chao1, ACE, and PD whole tree Beta: the microbial communities of the two groups were clustered separately and in close proximity
Watanabe et al., 2018 [45]	C57BL/6 mice	Male	7 weeks old	Not found	26% carbohydrate, 61% fat, 23% protein	4 weeks	Feces	Phylum: about 73% Firmicutes, 15% Bacteroidetes, 4% Proteobacteria, and 1% Actinobacteria Family: Lachnospiraceae, Peptostrept-ococcaceae, Clostridiaceae, Defemb-acteraceae, Helicobacteraceae, Porph-yromonadaceae, Rikenellaceae, Corio-bacteriaceae, Desulfovbrionaceae, Bcte-roidaceae, Peptococcaceae, Ruminoc-occaceae, Lactobacillaceae, and Streptococcaceae	Not found	Alpha: / Beta: the samples were closely clustered, and the microbiota composition between samples was similar
Zheng et al., 2018 [46]	C57BL/6 mice	Male	6 weeks old	70% carbohydrate, 10% fat, 20% protein	35% carbohydrate, 45% fat, 20% protein	5 months	Feces	Phylum: about 47% Firmicutes, 27% Bacteroidetes, 17% Proteobacteria, and 6% Actinobacteria Family: Coriobacteriaceae, Erysipel-otrichaceae, S24-7, Desulfovibrionaceae, Ruminococcaceae, Lachnospiraceae, Peptostreptococcaceae, Porphyromonadaceae, and Rikenellaceae	Increase: Bacteroidetes, Proteobacteria, Peptostreptococcaceae, Porphyromonadaceae, and Coriobacteriaceae Reduce: Firmicutes, Actinobacteria, Bifidobacteriaceae, and Lactobacillaceae	Alpha: Not found Beta: there is a certain distance between the two sets of sample points
Huang et al., 2021 [47]	Sprague-Dawley rats	Male	Not found	Standard chow diet (The Medical LaboratorymAnimal Center in Guangdong, China)	15% lard, 20% sucrose, 10% casein, 1.2% cholesterol, 0.2% sodium cholate, and 53.6% standard chow diet	9 weeks	Colon contents	Phylum: about 91% Firmicutes, 5% Proteobacteria, and 3.4% Bacteroidetes Genus: *Bacteroides*, *Allobaculum*, *Blautia*, *Lachnoclostridium*, *Parabacteroides*, *Staphylococcus*, *Fusicatenibacter*, *Shuttleworthia*, and *Ralstonia*	Increase: Firmicutes, Bacteroidetes, and *Ralstonia* Reduce: Proteobacteria, *Turicibacter*, *Acinetobacter*, *Brevundimonas*, and *Bacillus*	Alpha: Sobs, Shannon, Chao1, PD whole tree ↓ (*p* < 0.01); Goods coverage ↑ (*p* < 0.01) Beta: the respective aggregation areas do not overlap
Li et al., 2018 [48]	Sprague- Dawley rats	Male	200–220 g	Low-fat diet	80% low-fat diet feed + 10% egg yolk powder + 10% lard	8 weeks	Feces	Phylum: about 73% Firmicutes and 24% Bacteroidetes Genus: *Lactobacillus*, *Barnesiella*, *Prevotella*, *Pseudoflavonifractor*, *Lachnoclostridium*, *Flavonifractor*, *Desulfovibrio*, *Oscillibacter*, and *Ruminiclostriium*	Increase: *Lactobacillus*, *Barnesiella*, *Desulfovibrio*, and *Oscillibacter* Reduce: *Lachnoclostridium*, *Prevotella*, and *Pseudoflavonifractor*	Not found
Choi et al., 2017 [49]	ICR mice	Female	8 weeks old	Nomal chow diet	Containing 60% fat by energy	12 weeks	Mixed colon and cecal contents	Phylum: about 86% Firmicutes, 8% Actinobacteria, 7% Bacteroidetes Genus: *Lactobacillus*, *Akkermansia*, *Bacteroides*, *Prevotella*, *Ruminococcus*, *Rikenellaceae*, and *Dorea*	Increase: Firmicutes, Actinobacteria, *Lactobacillus*, and *Ruminococcus* Reduce: Bacteroidetes, *Prevotella*, and *Rikenellaceae*	Alpha: Not found Beta: the respective aggregation areas do not overlap
Zhao et al., 2017 [50]	Wistar rats	Male	160–180 g	70% carbohydrate, 10% fat, 20% protein	35% carbohydrate, 45% fat, 20% protein	10 weeks	Feces	Phylum: about 84% Firmicutes, 8% Bacteroidetes, and 2% Proteobacteria Genus: *Lachnoclostridium*, *Ruminococcaceae_UCG-014*, *Bacteroidales_S24-7_group_norank*, *Ruminococcaceae_UCG-005*, *Bilophila*, *Eubacterium_coprostanoligenes_group*, and *Akkermansia*	Increase: Firmicutes, *Lachnoclostridium*, and *Bilophila* Reduce: Bacteroidetes, Proteobacteria, *Ruminococcaceae_UCG-014*, *Bacteroidales_S24-7_group_norank*, *Ruminococcaceae_UCG-005*, *Eubacterium_coprostanoligenes_group*, and *Akkermansia*	Alpha: Shannon ↓ Beta: the respective aggregation areas do not overlap

Note: ↑ and ↓ represent higher (↑) or lower (↓) values in the HFD group compared with the CG group.

## Supplementary Materials

The following supporting information can be downloaded at: https://www.mdpi.com/article/10.3390/nu17091402/s1, Table S1: Effects of oral administration of a strain (belonging to the *Lactobacillus*) on mice fed a high-fat diet; Table S2: Effects of oral administration of a strain (belonging to the *Bifidobacterium*) on mice fed a high-fat diet; Table S3: Effects of oral administration of a strain (other probiotics/potential probiotic) on mice fed a high-fat diet.

## Abbreviations

The following abbreviations are used in this manuscript.

5-HT	5-hydroxytryptamine
ABCG	ATP-binding cassette transporters G
ACC	acetyl CoA carboxylases
ACO	1-aminocyclopropane-1-carboxylicacid oxidase
AML12	alpha mouse liver 12
AMPKα2	kinase AMP-activated catalytic subunit alpha 2
AP-1	activator protein-1
APOB48	apolipoprotein B48
ATGL	adipose triglyceride lipase
BA	bile acid
BSEP	bile salt export pump
BSH	bile salt hydrolase
C/EBP-α	CCAAT/enhancer binding protein alpha
CAT	catalase
CCL2	C-C motif ligand 2
CCR2	C-C chemokine receptor 2
CPT1α	carnitine palmitoyltransferase-1 alpha
CPTIα	carnitine palmitoyltransferase-I-alpha
CRP	C-reactive protein
CYP7A1	cholesterol 7α-hydroxylase
DCA	deoxycholic acid
DGAT1	diacylglycerol acyltransferase 1
F/B	Firmicutes/Bacteroidetes
FABP2	fatty acid binding protein 2
FAS	fatty acid synthase
FAT/CD36	fatty acid translocase
FATP4	fatty-acid transport protein 4
FFA	free fatty acid
FGF21	fibroblast growth factor 21
FXR	farnesoid X receptor
GLP-1	glucagon-like peptide 1
HDL-C	high-density lipoprotein cholesterol
HFD	high fat diet
HSL	hormone-sensitive triglyceride lipase
IFN-γ	interferon-gamma
IGF-1	insulin-like growth factor-1
IGFBP-3	insulin-like growth factor binding protein-3
IL-1	interleukin 1
IL-6	interleukin 6
LCA	lithocholic acid
LDL-C	low-density lipoprotein cholesterol
LDLR	low-density lipoprotein receptor
LEfSe	linear discriminant analysis effect size
LEP	leptin
LPL	lipoprotein lipase
LPS	lipopolysaccharide
LXRα	liver X receptors alpha
MAPK	mitogen-activated protein kinase
MCP-1	monocyte chemoattractant protein-1
MOGAT1	monoacylglycerol O-acyltransferase 1
MT-Cytb	mitochondrially encoded cytochrome b
NAFLD	nonalcoholic fatty liver disease
NF-κB	nuclear factor-kappaB
NRF1	nuclear respiratory factor 1
PGC1-α	PPAR-gamma co-activator-1 alpha
PI3K	phosphatidylinositol 3-kinase
PKA	protein kinase A
PLIN	perilipin
PPARα	peroxisome proliferator-activated receptor alpha
PPARγ	peroxisome proliferator-activated receptor gamma
PYY	peptide tyrosine tyrosine
RIP140	receptor-interacting protein 140
SCD-1	stearoyl-CoA desaturase-1
SCFAs	short-chain fatty acids
SNS	sympathetic nervous system
SOD1	superoxide dismutase 1
SREBP-1	sterol regulatory element-binding protein 1
TC	total cholesterol
TG	triglyceride
TGR5	takeda G protein-coupled receptor 5
TLR4	toll-like receptor 4
TNF-α	tumor necrosis factor-alpha
TRAF2	tumor necrosis factor receptor-associated factor 2
UCP1	uncoupling protein 1
ZO-1	zonula occludens-1
