# Supplementary material for: Microbiota-Based Intervention Alleviates High-Fat Diet Consequences Through Host-Microbe Environment Remodeling
Source: Nutrients. 2025 Apr 22;17(9):1402. doi: 10.3390/nu17091402 (PMC12073166; doi:10.3390/nu17091402)
Supplement: Supplementary file 1 [file nutrients-17-01402-s001.zip › nutrients-3589964-supplementary.pdf]

Supplementary table S1 Effects of oral administration of a strain (belonging to the *Lactobacillus*) on mice fed a high-fat diet

Refer-ences	Speci-es	Sex	Age or weight	Probiotic/P-otential Probiotic	Resouces	Bacterial concentrat ion	Treatm-ent cycle	Body weight and serum biochemical indicators	Liver	Adipose tissue	Intestinal	Gut microbe	SCFA and BA content
Chen et al., 2020 [65]	Kun-ming mice	Male	6 weeks old, 20 ± 2 g	*Lactiplanti-bacillus plantarum* FZU3013	Isolated from the traditional brewing process of Hongqu rice wine	1× 10^9 CFU/mL	8 weeks	body weights↓<br>epididymal fat index↓<br>the size of the adipocytes↓<br>serum TG, TC and LDL-C levels↓<br>HDL-C/LDL-C value ↑	the levels of TG, TC, NEFA and BAs↓<br>the size and number of lipid droplets↓<br>BSEP, FXR, CYP7A1, NTCP, LDLR and ACOX1 mRNA expression levels ↑<br>HMGCR, FAS, FATP, ACC1, ACAT2,C/EBP-α,CD36 and SREBP-1c mRNA expression levels ↓<br>CD36 and SREBP-1C protein expression↓<br>CYP7A1 protein expression ↑	not found	not found	*Flavonifractor*, *Alistipes*, *Ruminococcus*, *Anaerovorax*, *Helicobacter* and *Odoribacter*↑<br>*Desulfovibrio* ↓	fecal BAs level↑
Sun et al., 2023 [66]	ICR mice	Male	32 ± 4 g	*Lactiplanti-bacillus plantarum* NKK20	Zhenjiang Tianyi Biotechno logy Co., Ltd. (China)	1×10^9 CFU / animal / day	7 weeks	body weights↓<br>plasma concentrations of TC and TG ↓	the mRNA expression of CYP7A1 ↑, CTGF and TGF-1 ↓<br>degree of adipose degeneration in hepatocytes ↓	not found	not found	*Pseudomonas* and *Turicibacter* ↓<br>*Akkermansia* ↑	acetic acid, propionic acid, and butyric acid concentrations ↑<br>the concentrations of cholic acid, glycinocholic acid, and glycinodeoxycho-lic acid in the livers ↓<br>the concentration of aminodeoxycholic acid ↑
Jacout on et al., 2023 [67]	C57B-L/6J	Male	6-8 weeks old	*Lactiplanti-bacillus plantarum* CGMCC	isolated from the feces of a healthy child and kindly provided by Indigo Therapeut ics	1 × 10^9 CFUs / animal / day	12 weeks	fasting glucose levels↓<br>Serum levels of LDL-c ↓	G6PASE and GLUT-4 mRNA expression levels ↓<br>FASN mRNA expression levels ↓<br>PPAR-α mRNA expression levels ↑	not found	the expression of ZO-1 protein↑	ratio of Firmicutes to Bacteroidetes↓<br>*Alloprevotella*, *Lactobacillus*, *Parasutterella*, and *Acinetobacter* ↑	concentrations of valerate ↓
Cai et al., 2020	Kun-ming mice	female	7 weeks old	*Lactiplanti-bacillus plantarum*	isolated from Poland	2 × 10^8 CFUs / animal /	8 weeks	body weight↓<br>lipid accumulation↓	liver weight ↓<br>TG concentration↓<br>SREBP-1 and DGAT1	not found	not found	Firmicutes/Bacteroid-etes ↓<br>*Bacteroides*, *Alistipes*,	not found

[68]				FRT10	sour dough	day			mRNA expression levels↓<br>PPAR-α mRNA expression level↑			*Intestinimonas*, *Butyricicoccus*, *Odoribacter*, *Butyricimonas*, and *Oscillibacter*↑<br>*Roseburia*, *Blautia*, and *Lachnoclostridium*↓	
Jang et al., 2024 [69]	C57B-L/6 mice	male	7 weeks old	*Lactiplanti-bacillus plantarum* NCHBL-004	isolated from honeybees (collected in Gwangju, Republic of Korea)	1 ×10^8 CFU / animal / day	12 weeks	body weight↓<br>plasma-active GLP-1↑	liver weight ↓<br>TG and TC concentration↓<br>lipid accumulation↓<br>C/EBPα, FABP4, ACC,IL-1β、TNF-α , IL-6 and MCP-1 mRNA expression levels ↓	not found	not found	*Lactobacillus* ↑	acetic acid and propionic acid concentrations in feces↑<br>BAs concentration in feces↑
Gan et al., 2020 [70]	C57B-L/6 mice	half male and half female	6 weeks old	*Lactiplanti-bacillus plantarum* CQPC01	isolated from Sichuan Paocai in Chongqing, China	1 × 10^9 CFU/kg body weight	6 weeks	body weight↓<br>Serum levels of TG, TC and LDL-c↓<br>Serum levels of HDL-c↑<br>serum levels of IL-6, IL-1β, TNF-α and IFN-γ↓<br>serum levels of IL-4 and IL-10 ↑	Liver index↓<br>levels of TG, TC and LDL-c↓<br>levels of HDL-c↑<br>C/EBP-α and PPAR-γ mRNA expression levels↓<br>CYP7A1, CPT1, LPL, CAT, SOD1, and SOD2 mRNA expression levels↑	the size of adipocytes↓	not found	not found	not found
Choi et al., 2023 [71]	C57B-L/6J mice	male	6 weeks old	*Lactiplanti-bacillus plantarum* SKO-001	isolated from Angelica gigas Nakai	5 × 10^9 CFU/day, 1 × 10^10 CFU/day and 2 × 10^10 CFU/day	12 weeks	body weight↓<br>Serum levels of adiponectin↑ and leptin↓<br>Serum levels of TC, LDL-C, FFA and TG↓	lipid accumulation ↓<br>SREBP-1c and PPARγ mRNA expression and protein levels↓	average adipocyte size in white adipose tissues (subcutaneous, visceral, and epididymal) ↓<br>UCP-1 mRNA expression level↑<br>PPARγ, C/EBPα and SREBP-1c mRNA expression levels ↓	not found	not found	not found
Choi et al., 2020 [72]	C57B-L/6-N mice	male	5 weeks old	*Lactiplanti-bacillus plantarum* LMT1-48	isolated from the traditional Korean fermented food kimchi	1 × 10^9 CFU/day	16 weeks	body weight↓<br>the plasma levels of leptin↓	liver weight ↓<br>levels of TG↓<br>PPARγ, HSL, SCD-1 and CD36 mRNA expression levels ↓	the epididymal fat pad weight↓<br>the size of visceral adipocytes↓	not found	not found	not found

Gan et al., 2020 [73]	C57B-L/6 mice	half male and half female	6 weeks old	*Lactiplanti-bacillus plantarum* CQPC03	isolated from Sichuan Paocai in Chongqin g, China	1 × 10^9 CFU/kg body weight	8 weeks	body weight↓<br>serum IL-10 and IFN-γ ↓, IL-4↑	liver weight ↓<br>SOD and GSH-Px↑, MDA↓<br>CPT1, LPL, CAT, and SOD1 mRNA expression levels↑	epididymal fat weight ↓	not found	not found	not found
Yin et al., 2020 [74]	C57B-L/6J mice	male	5 weeks old	*Lactiplanti-bacillus plantarum* Shinshu N-07	Isolation of lactic acid bacteria from fermented Brassica rapa	1×10^10 CFU/g	8 weeks	body weight↓	total lipid levels ↓<br>ACC1 and PPARγ mRNA expression levels ↓	epididymal adipose tissues↓<br>cross-sectional area of adipocytes↓<br>UCP1 mRNA expression levels↑	not found	*Lactobacillaceae* and *f_S24-7*↑	not found
Liu et al., 2020 [75]	C57B-L/6 mice	male	6 weeks old	*Lactiplanti-bacillus plantarum* Y44	Dalian Probiotics Function Research Key Laborato-r y	4×10^7 CFU/mL, 4×10^9 CFU/mL	12 weeks	body weight↓<br>serum TC, TG and LDL-C levels↓<br>serum HDL-c ↑	liver weight ↓<br>FAS and ACC protein levels↓	epididymal fat and perirenal fat ↓<br>epididymal adipocyte area↓	the expression of both p38 and p-p38 in colon↓<br>the protein expression of occludin and claudin-1↑	the ratio of Firmicutes/Bacteroide tes↓<br>Bacteroidaceae, Muribaculaceae, Lactobacillaceae and Rikenellaceae↑<br>Ruminococcaceae and Desulfovibrionaceae ↓	propionic acid, butyric acid, butanoic acid-3-methyl, pentanoic acid and acetic acid↑
Chen et al., 2023 [76]	C57B-L/6J mice	male	4 weeks old	*Lactiplanti-bacillus plantarum* HF02	isolated and identified from Qula cheese made from yak milk	2 × 10^8 CFU/mL/ day, 2 × 10^9 CFU/mL/ day	8 weeks	body weight↓<br>serum TG, TC, LDL-C, FFAs levels↓<br>serum HDL-c level ↑<br>serum LPS, IL-1β and TNF-α↓	liver index↓<br>TG, TC and FFAs level↓<br>lipid droplet field↓<br>cell size of epididymal adipose↓<br>the mRNAexpression levels of AMPK, PPARα, CPT-1a, and ACOX1↑<br>the ratio of p-AMPK/AMPK and PPARα, CPT-1a, and ACOX1 protein levels↑	epididymal and perirenal adipose index↓	not found	Ratio of Bacteroidetes/Firmicu tes↓<br>Muribaculaceae, *Rikenella-ceae_RC9_ gut_group*, and *Faecalibaculum*↑<br>*Bacteroides*, *Alistipes*, *Blautia*, and *Colidextribacter*↓	SCFAs (acetic acid, pro-pionic acid, and butyric acid) levels↑
Das et al., 2024 [77]	albino mice	male	13.59 ± 0.56 g	*Lactiplanti-bacillus plantarum* E2 MCC-KT	isolated from a fermented beverage	1×10^9 CFU/mL	8 weeks	body weigh↓<br>serum TG, TC, LDL-c levels↓<br>serum HDL-c level ↑	fat deposition and droplets↓<br>PPARα, ACO and CPT1 protein levels ↑<br>SREBP-1c, ACC, FAS mRNA expression levels ↓	PPAR-α and IL10 protein levels ↑<br>leptin receptor TNF-α and IL-1Ra protein levels ↓	not found	not found	not found
Ghosh et al., 2024 [78]	Swiss albino mice	male	6 weeks old	*Lactiplanti-bacillus plantarum* KAD	isolated from ethnic fermented food 'Kinema' found in Darjeeling , India	2×10^10 CFU/mL	8 weeks	body weigh↓<br>Serum Insulin Level↓<br>serum TG, TC, LDL-c, VLDL-c levels↓, HDL-C level↑<br>serum TNF-α and IL-6 protein	liver Co-efficient↓<br>steatosis, immune infiltration, swollen hepatocytes with distorted histology↓<br>TNF-α and IL-6 protein levels↓	pgWAT Co-efficient↓<br>IL-6 protein level↓	Intestine Co-efficient↓<br>SOD, GSH and CAT enzymes levels↑, MDA level ↓<br>TNF-α and IL-6 protein	Prevotellaceae, Lactobacillaceae, *Prevotella*, *Lactobacillus*, unclassified Lachnospiraceae↑<br>*Unclassified Bacteroidales*↓	not found

								levels↓			levels↓		
Cai et al., 2025 [79]	C57B-L/6J mice	male	4 weeks old	*Lactiplanti-bacillus plantarum* BXM2	isolated from a naturally fermented passion fruit honey beverage in the Fujian province, China	2 × 10^9 CFU / animal / day	6 weeks	body weigh not statistically significant	liver index not statistically significant	perirenal fat index↓	villus height to crypt depth↑ the number of goblet cells↑ TNF-α mRNA expression level↓, IL-6 level ↑	Firmicutes/Bacteroidota ratio↓ *Dubosiella*, *Romboutsia* and *Lachnospiraceae_UCG-006*↓ *Akkermansia* and *Lactobacillus*↑	not found
Lee et al., 2024 [80]	C57B-L/6 mice	male	4–6 weeks old	*Lactiplanti-bacillus plantarum* DSR330	isolated from kimchi in Korea	1×10^8 CFU/animal/day, 1×10^9 CFU/ animal/ day	7 weeks	body weigh↓ serum TG, TC, LDL-c, ASTand ALT↓ serum Insulin and Leptin levels↓, Adiponectin level ↑	lipid droplets↓ PPARγ, SREBP-1c and FAS mRNA expression levels↓, ACC1, ACO, PPARα and CPT-1 levels ↑	Subcutaneous fat↓	not found	not found	not found
Soundharrajan et al., 2020 [81]	ICR mice	male	7 weeks old	*Lactiplanti-bacillus plantarum* KCC48	isolated from alfalfa (Cheonan, South Korea)	1×10^9 CFU/ animal/ day	8 weeks	body weigh↓ serum ALT, AST, TC, TG and LDL levels↓, HDL, CAT, SOD and GSH levels↑	ALT and AST levels↓	adipose tissue mass↓ adipose tissue IL1β, IL6, TNFα, and leptin mRNA expression↓ epididymal fat tissues PPAR-γ, C/EBP-α, SREBP-1,ACC, FAS, and aP2 protein levels↓, Adiponectin level↑	not found	*Lactobacillus*↑	not found
Ondee et al., 2021 [82]	C57B-L/6 mice	male	8 weeks old	*Lactiplanti-bacillus plantarum* dfa1	Thai-isolated probiotics	1×10^8 CFU	16 weeks	body weigh↓ serum TC and TG levels↓ TNF-α, IL-6 and IL-10 levels↓	liver weight↓ liver cholesterol↓ GSH level↑, MDA level ↓	mesentery, peri-renal, retro-peritoneum, peri-gonadal, and subcutaneous fat↓	TNF-α and IL-6 levels↓ enterocyte IL-8 production↓	Bacteroides↑, Proteobacteria↓	not found
Lee et al., 2018 [83]	C57B-L/6J mice	male	12 weeks old	*Lactiplanti-bacillus plantarum* strain Ln4	isolated from napa cabbage	5 ×10^8 CFU /animal/ day	5 weeks	body weigh↓ plasma insulin and TG levels↓ epidydimal fat ANGPT-L3, CRP, leptin, lipocalin-2, MCP-1 and IGFBP protein	liver weight↓ IRS2, Akt2, LPL and AMPK mRNA expression levels↑, CD36 level↓	lipid accumulation↓ epidydimal fat and brown adipose tissue↓	not found	not found	not found

								levels ↓					
Riezu-Boj et al., 2022 [84]	C57B-L/6 mice	male	8 weeks old	*Lactiplanti-bacillus plantarum* DSM20174	isolated from a pickled cabbage from the Spanish Collection of Type Cultures	1 × 10^9 CFU / animal / day	10 weeks	body weigh↓<br>serum TG, TC, and NEFAs ↓	IL6, TLR4, and MCP1 mRNA expression levels↓	the number of crown-like structures of WAT ↓<br>F4/80, MCP1, CD11c, CD68 and CD44 mRNA expression levels↓<br>PPARγ and SREBP mRNA expression levels ↑	not found	*Ruminococcus torquesx*↓<br>*Lactiplantibacillus plantarum* spp. ↑	not found
Huang et al., 2021 [85]	C57B-L/6J mice	male	5 weeks old	*Lactiplanti-bacillus plantarum* KC28	isolated from kimchi produced in the Pohang region (South Korea)	5 ×10^9 CFU / animal / day	12 weeks	body weigh↓<br>serum TC, TG and LDL-c levels↓	liver weight↓<br>PPAR-α, PGC1-α, CPT1-α mRNA expression levels↑	adipose tissues weight↓<br>mesenteric adipose tissue (MAT) ACOX-1、PPAR-γ and FAS mRNA expression levels ↓ ,<br>PPAR-α, PGC1-α, CPT1-α mRNA expression levels↑	not found	Desulfovibrionaceae and Lactobacillaceae↑	Butyrate↑
Kuerman et al., 2021 [86]	C57B-L/6J mice	male	4 weeks old	*Lacticasei-bacillus paracasei* S0940<br>*Streptococ-cus thermophil-us* ldbm1	isolated from a traditional Chinese fermentation<br>isolated from traditional fermented yogurt	1 × 10^8 CFU/day,<br>1 × 10^9 CFU/day,<br>1 × 10^10 CFU/day	6 weeks	body weigh↓<br>serum TC, TG and LDL-c↓,<br>HDL-c ↑	TC and MDA levels↓<br>the contents of GSH-PX, CAT and MDA↑	not found	not found	*Lactobacillus* ↑	not found
Li et al., 2016 [87]	ICR mice	not found	7 weeks old	*Lactobacill-us casei* IBS041<br>*Lactobacill-us acidophilus* AD031<br>*Bifidobacte-rium bifidum*	Bifido Co., Ltd. (Hongchun, Korea)	5×10^8 CFU/mL	8 weeks	body weigh↓<br>*Lactobacillus acidophilus* AD031 and *Bifidobacterium bifidum* BGN4 serum ALT and AST level↓	*Lactobacillus acidophilus* AD031 and *Bifidobacterium longum* BORI liver weight ↓<br>*Lactobacillus acidophilus* AD031 and *Bifidobacterium bifidum* BGN4 TG level ↓<br>*Bifidobacterium longum* BORI TC level ↓	adipocyte sizes↓	not found	not found	not found

				BGN4<br>*Bifidobacterium longum* BORI									
Liu et al., 2022 [88]	C57BL/6J mice	male	7-8 weeks old	*Lacticaseibacillus paracasei* 24	isolated and screened in the intestines of healthy adults	1×10^8 CFU / animal / day	8 weeks	serum TC, TG, LDL-c, ALT, AST, LPS, LEP and NEFA↓, HDL-c↑	TC, TG, NEFA and MDA levels ↓<br>CAT, SOD and GSH-Px↑	Adipose tissue FAS, ACC, SCD-1 and SREBP-1c expression levels↓, AMPK-α,CPT-1α, PPARα and HSL levels ↑	not found	Firmicutes/Bacteroidota ratio↓<br>Proteobacteria ↓<br>*Akkermansia* ↑	not found
Miao et al., 2023 [89]	C57BL/6J mice	male	6 weeks old	*Lacticaseibacillus paracasei* K56	Yili Industrial Group Co., Ltd	1×10^7, 1×10^9, and 1×10^11 CFU/day	10 weeks	body weigh↓<br>Fasting plasma glucose, serum insulin↓	FAS protein level↓,<br>PPARγ level ↑	perirenal and epididymal fat mass↓<br>the fat area of abdominal and subcutaneous adipose tissues↓	not found	*Coriobacteriaceae_UCG-002*, *Desulfovibrio* and *Dubosiella* ↓	not found
Miao et al., 2022 [90]	BALB/c mice	not found	Neonatal mice	*Lacticaseibacillus paracasei* N1115	Shijiazhuang Junlebao Dairy Co. Ltd., Shijiazhuang, China P.R.	1×10^9 CFU	15 weeks	serum levels of insulin and leptin↓	liver weight↓	visceral fat↓	not found	*Bilophila*, *Lachnoclostridium*, and *Blautia* ↑	SCFAs concentration↑
Galindev et al., 2024 [91]	C57BL/6J mice	male	3 weeks old	*Lacticaseibacillus paracasei* X-1<br>*Lacticaseibacillus paracasei* X-17	isolated from Mongolian fermented dairy products	5 × 10^9 CFU/day	6 weeks	body weight↓<br>Fasting plasma glucose↓<br>serum TG and LDL levels ↓	not found	epididymal fat index↓	not found	not found	not found
Lee et al., 2024 [92]	C57BL/6J mice	male	6 weeks old	*Lacticaseibacillus paracasei* BEPC22<br>*Lactiplantibacillus plantarum* BELP53	isolated from Korean faeces	2 × 10^9 CFU / animal / day	10 weeks	body weight↓<br>Serum Ghrelin level↑	PPARγ and UCP2 mRNA expression levels ↓	adipocyte mean area↓<br>PPARα mRNA expression level ↑, UCP2 level↓	not found	*Colidextribacter* and *Tuzzerella*↓<br>*Alistipes*↑	not found
Lv et al., 2021 [93]	Kunming mice	male	6 weeks old	*Lacticaseibacillus paracasei* FZU103	isolated from traditional brewing of Wuyi	1 × 10^9 CFU / animal / day	8 weeks	body weight↓<br>serum TC and LDL-c levels ↓,<br>HDL-c level ↑	accumulation of lipid droplets↓<br>ACOX1, FXR, NTCP mRNA expression levels↑<br>FAS, ACAT2, FATP,	epididymal white adipose tissue↓<br>the size of epididymal	not found	*Ruminococcus*, *Alistipes*, *Pseudoflavonifractor* and *Helicobacter*↑<br>*Blautia*,	excretion of BAs through feces↑

					Hong Qu glutinous rice wine				SREBP-1c mRNA expression levels↓	adipocytes↓		*Staphylococcos* and *Tannerella*↓	
Song et al., 2023 [94]	C57B-L/6 mice	male	6 weeks old	*Lacticasei-bacillus paracasei* AO356	isolated from fecal samples of healthy Koreans	5×10^7 CFU/mg	10 weeks	body weight↓<br>serum glucose, insulin and HOMA-IR levels↓	not found	fat accumulation↓	not found	Bacteroidaceae↓	not found
Novotny et al., 2015 [95]	BALB/c mice	female	5 weeks old	*Lactobacill-us casei* CRL 431	obtained from the CERELA Culture Collection (San Miguel de Tucumán, Argentina)	2×10^8 CFU/mL	8 weeks	not found	CD4+ level ↓, F4/80+ level ↑<br>IL-6 protein level↑, TNF-α and IL-17 levels ↓	IL-6, IL-10 and MCP-1 protein levels↓	IFN-γ protein level↓, IL-10↑	not found	not found
Nguyen et al., 2022 [96]	mice	not found	not found	*Lactobacill-us sakei* MJM60958	isolated from fermented food	1×10^8 CFU, 1×10^9 CFU	12 weeks	body weight↓<br>serum TG, leptin, ALT, AST, TNF-α, IL-1β and UA levels ↓, BUN and adiponectin levels↑	Liver weight and liver weight/body weight↓<br>the fat content↓<br>FAS, ACC mRNA expression levels↓, PPARα, CPT1A mRNA expression levels ↑<br>FAS and SREBP-1 protein levels↓, PPARα protein levels↑	not found	not found	Firmicutes, Actinobacteria, *Clostridiales vadinBB60 group*, *Clostridiaceae 1*, Atopobiaceae, Lachnospiraceae, and Peptostreptococcaceae ↓<br>Venrrucomicrobia, Tenericutes, Akkermansiaceae and Ruminococcaceae ↑	acetic acid ↑
Chen et al., 2024 [97]	C57B-L/6J mice	male	3 weeks old	*Latilactoba-cillus sakei* QC9	isolated from traditional Chinese fermented food	1.0×10^9 CFU/mL	12 weeks	body weight↓<br>Fasting plasma glucose↓<br>serum TC, TG, LDL-c, TNF-α, IL-1β and IL-6 levels ↓, HDL-c, GSH and IL-10 levels ↑	TC and TG level↓<br>SOD and CAT ↑, MDA ↓	not found	not found	*Lactobacillus* and Bacteroidetes↑	butyrate↑
Park et al., 2021 [98]	C57B-L/6 mice	male	6 weeks old	*Latilactoba-cillus sakei* WIKIM31	isolated from homemade kimchi (Gangwon province, Republic of Korea)	1×10^9 CFU / animal / day	12 weeks	body weight↓<br>serum TG, TC, glucose, Leptin and resistin levels↓<br>serum adiponectin level ↑	lipid droplet↓<br>PPARγ, C/EBPα, FAS and CD36 mRNA expression levels ↓<br>UCP2 and CPT-1α mRNA expression levels ↑<br>TNF-α, IL-6, and MCP-1 mRNA expression levels↓	fat mass (epididymal and abdominal adipose tissue)↓<br>adipocyte size↓<br>epididymal fat PPARγ, C/EBPα, FAS and CD36 mRNA expression	TNF-α, IL-6, and MCP-1 mRNA expression levels↓	not found	propionate, butyrate, valerate↑

										levels↓<br>epididymal fat UCP2 and CPT-1α mRNA expression levels↑			
Lim et al., 2016 [99]	C57B-L/6J mice	male	5 weeks old	*Lactobacillus sakei* OK67	isolated from kimchi	1 × 10^9 CFU / animal / day	17 weeks	body weight↓<br>blood LPS levels ↓	not found	epididymal fat↓<br>adipose tissue PPARγ, FAS, and αFABP mRNA expression level ↓	TNF-α, IL-1β, IL-6, iNOS, COX-2 and NF-κB protein level ↓<br>IL-10, Arg1, CD204, tight junction proteins level ↑	not found	not found
Jang et al., 2019 [100]	C57B-L/6 mice	male	6 weeks old	*Lactobacillus sakei* OK67<br>*Lactobacillus sakei* PK16	not found	2 × 10^9 CFU / animal / day	9 weeks	body weight↓<br>serum TG, TC, and LPS levels↓	liver weight↓<br>serum TG, TC, and LPS levels↓<br>AMPK protein and SIRT1, PGC-1α, and Fiaf mRNA expression levels↑<br>SREBP-1c, LPL, G6PD, and FAS mRNA expression↓	epididymal fat pad weight↓	colon shortening↓<br>TNF-α mRNA expression level↓, IL-10 level↑	Proteobacteria and Firmicutes populations,Proteobacteria/Bacteroidetes↓	not found
Won et al., 2020 [58]	C57B-L/6J mice	male	5 weeks old	*Lactobacillus sakei* ADM14	isolated from Andong sik-hae kimchi	10^8-10^9 CFU	10 weeks	body weight↓<br>fasting blood glucose↓<br>serum TC, LDL-c and HDL-c levels↓	not found	epididymal fat mass↓<br>epididymal fat pad PPARγ, C/EBPα,CD36 and MCP-1 mRNA expression levels↓	not found	Bacteroidetes, Deferribacteres, Bacteroidia, Clostridia, Deferribacteres, Bacteroidaceae, Lachnospiraceae, and Muribaculaceae↑<br>Verrucomicrobia, Erysipelotrichia↓	butyrate↑
Ji et al., 2019 [101]	C57B-L/6 mice	male	4 weeks old	*Lactobacillus sakei* CJLS03	isolated from Korean fermented kimchi	1 × 10^9 CFU / animal / day	8 weeks	body weight↓<br>serum TG, FFA and leptin level↓	not found	mesenteric, epididymal and subcutaneous adipose tissues↓<br>Subcutaneous and mesenteric adipose tissue sizes↓<br>epididymal adipose tissue MCP1, IL-1β, SREBP-1c, FAS and SCD1 mRNA expression levels ↓ , IL-10	not found	*Lactobacillus* spp.↑	propionate and butyrate ↑

										level ↑<br>epididymal adipose tissue AMPK protein level ↑			
Kang et al., 2022 [102]	C57B-L/6 J mice	male	3 weeks old	*Lactobacill-us acidophilus*	isolated from porcine guts	5×10^9 CFU/day	12 weeks	body weight↓<br>serum TC, TG and HDL levels ↓	liver weight↓<br>lipid area, LPS ↓<br>TNF-α, IL-1β, F4/80, NF-κB, TLR4, IκB and P-IκB protein levels ↓<br>Leptin mRNA expression level↑,PPARγ level↓	epididymal white adipose tissue↓<br>UCP-1 expression level ↓<br>TNF-α and IL-1β protein levels↓<br>CPT-1, Leptin, HSL mRNA expression levels↑<br>FAS and PPARγ mRNA expression levels ↓	TNF-α and IFN-γ protein levels ↓	*Lactobacillus*, *Bifidobacterium*, *Bifidobacterium pseudolongum*, *Lactobacillus johnsonii*↑	not found
Tang et al., 2021 [103]	Kun-ming mice	male	4 weeks old	*Lactobacill-us acidophilus* NX2-6	not found	10^9 CFU/mL	17 weeks	body weight↓	PGC-1α, SIRT1, nrf1, p-AMPKα, GLUT2, PI3K, p-AKT, Glucokinase and FGF21 protein levels ↑<br>PEPCK, Ser307, p50, p-p65, p-p38, p-JNK, p-ERK protein levels↓	PEPCK, p50, p-p38 and Ser307 protein levels↓<br>GLUT2, PI3K, p-AKT, Glucokinase, p-AKTand Glucokinase protein levels↑	PEPCK, GLUT2, Glucokinase protein levels↑	not found	not found
Song et al., 2015 [104]	C57B-L/6 mice	male	7 weeks old	*Lactobacill-us acidophilus* NS1	isolated from infant feces	1.0 × 10^8 CFU/mL	10 weeks	body weight↓<br>serum TG, TC, LDL-c levels ↓	TC and TG level↓<br>SREBP2 and LDLR mRNA expression level↑	not found	not found	not found	not found
Ondee et al., 2021 [105]	C57B-L/6 mice	male	8 weeks old	*Lactobacill-us acidophilus* LA5	Chr. Hansen, Hørsholm, Denmark	1 × 10^8 CFU/day	8 weeks	body weight↓<br>serum TG, TC, HDL, LDL, TNF-α, IL-6, and IL-10 ↓	not found	mesentery, retro-peritonium , peri-gonadal, peri-renal and subcutaneous fat ↓	ZO-1 and MUC2 protein levels↑	Firmicutes, Verrucomycobia, Cyanobacteria, *Akkermansia muciniphila*↑<br>Gammaproteobacteria ↓	not found
Zheng et al., 2024 [106]	mice	not found	not found	*Lactobacill-us acidophilus* GOLDGU-TLA100	not found	not found	not found	body weight ↓<br>serum glucose and cholesterol level↓	lipid accumulation↓	adipose inflammation↓	not found	not found	not found
Molin a-Tije ras et	C57B-L/6 J mice	male	5 weeks old	*Lactobacill-us fermentum*	Biosearch, S. A. (Granada,	5 × 10^ 8 CFU / animal /	11 weeks	body weight ↓<br>plasma Gluose, HOMA-IR,	IL-6, TNF-α, MCP-1, JNK-1, TLR4 mRNA expression levels ↓	abdominal fat and epididymal fat ↓	MUC-1, MUC-2, MUC-3,	*Bacteroides*, *Akkermansia* sp.↑<br>Erysipelotrichi class	not found

al., 2021 [107]				CECT5716	Spain)	day		LDL-c, TG levels↓	GLUT4, Leptin-R mRNA expression level↑	adipocytes area↓<br>fat IL-6, TNF-α, MCP-1, JNK-1, TLR4, Leptin mRNA expression levels ↓<br>fat PPARα, GLUT4, AMPK, Adiponectin, Leptin-R mRNA expression levels↑	ZO-1, Occludin and TFF-3 mRNA expression levels↑<br>Occludin protein level↑<br>LPS level↓	and *Clostridium* spp↓	
Li et al., 2024 [108]	C57B-L/6 J mice	male	6 weeks old	*Limosilact-o bacillus fermentum* HNU312	isolated from fermented Yucha (a traditional fermented food from Hainan Province, China)	1 × 10^ 8 CFU / animal / day	10 weeks	body weight↓<br>serum TG, TC, LDL-c, HDL-c, IL-6 and TNF-α levels↓<br>blood glucose, insulin levels↓	liver weight↓<br>lipid droplet area ↓	epididymal fat weight↓<br>adipocyte size↓	not found	*Akkermansia muciniphila* and *Bacteroides xylanisolvens*↑<br>*Enterorhabdus caecimuris*↓	acetic acid, propionic acid, butyric acid, isobutyric acid, valeric acid, isovaleric acid concentrations ↑
Kim et al., 2021 [109]	C57B-L/6J mice	male	4 weeks old	*Limosilact-o bacillus fermentum* MG4231<br>*Limosilact-o bacillus fermentum* MG4244	Mediogen Co., Ltd. (Jecheon, Korea)	2×10^8 CFU / animal / day	8 weeks	body weight ↓<br>plasma ALT, TC, LDL, Leptin levels↓<br>the combination of MG4231 and MG4244 group plasma Adiponectin level ↑	TG level↓<br>SREBP-1c, FAS protein levels↓<br>p-AMPK, p-ACCprotein leves ↑	adipose tissue (liver, epididymal and subcutaneous) weight↓<br>the combination of MG4231 and MG4244 group adipocyte diameter↓<br>epididymal adipose tissue (MG4244 and combination group) PPARγ, FAS, aP2 protein levels↓	not found	not found	not found
Lee et al., 2023 [110]	C57B-L/6 mice	male	5 weeks old	*Limosilact-o bacillus fermentum* MG4294<br>*Lactiplanti-bacillus plantarum* MG5289	MEDIOG EN Co., Ltd. (Jechon, Republic of Korea)	1 × 10^9 CFU / animal / day	12 weeks	body weight↓<br>serum AST, ALT, TC, LDL-c levels↓	liver weight↓<br>lipid droplets↓<br>TG and TC levels↓<br>PPARγ, C/EBPα, p-AMPK, m-SREBP1, and FAS protein levels ↓	not found	TNF-α,IL-1β and IL-6 protein levels↓	not found	not found

Yoon et al., 2020 [111]	C57BL/6N mice	male	6 weeks old	*Lactobacillus fermentum* LM1016	obtained from Lactomason (South Korea)	1 × 10^9 CFU / animal / day	8 weeks	body weight↓<br>fasting blood glucose, fasting serum insulin, leptin and TC levels↓	liver weight↓<br>lipid droplet size and TG accumulation↓<br>CYP7A1 and CYP27A1 mRNA expression levels↑, FGF15, GCK, PEPCK, G6PASE, PPARγ, SREBP1, ACC, FAS, SCD1, ACACB, DGAT2, CHERBP, CIDEC, G0S2 levels ↓	brown adipose tissue lipid droplet sizes↓<br>Inguinal white adipose tissue and gonadal white adipose tissue weight ↓<br>brown adipose tissue UCP1, DIO2, ACADM, ESRR, and AOX mRNA expression levels↓<br>gonadal white adipose tissue F4/80, Mcp1, TGF-β, IFN-γ, IL-1β, and IL-18 mRNA expression levels↓,TNF-α signaling via nuclear factor NF-κB↓	not found	not found	serum bile acid levels↑
Song et al., 2023 [112]	C57BL/6 J mice	male	6 weeks old	*Lactobacillus fermentum* CKCC1858<br>*Lactobacillus fermentum* CKCC1369<br>*Lactiplantibacillus plantarum* CKCC1312<br>*Lactobacillus gasseri* CKCC1913	isolated from infant feces<br>isolated from fermented bamboo shoot<br>isolated from pickles<br>isolated from goat milk curd	5 × 10^10 CFU/kg of body weight	7 weeks	fasting blood glucose levels↓	TG, TC, LDL-C, FFAs, SOD, GSH, and CAT levels↓<br>MDA, IL-6 and TNF-α levels ↓	not found	not found	Firmicutes/Bacteroidetes ratio↓<br>*Lactobacillus fermentum* CKCC1858: *Lactobacillus*↑, *Faecalibaculum*↓<br>*Lactobacillus fermentum* CKCC1369: *Bifidobacterium* and *Dubosiella*↑, *Faecalibaculum*↓<br>*Lactiplantibacillus plantarum* CKCC1312: *Lactobacillus*, *Prevotella* and *Akkermansia*↑, *Faecalibaculum*↓<br>*Lactobacillus gasseri* CKCC1913: *Lactobacillus* and *Prevotella*↑,	not found

												Faecalibaculum↓	
Schmidt et al., 2024 [113]	C57B-L/6 mice	male	3 weeks old	*Lacticasei-bacillus rhamnosus* LB1.5	isolated from raw buffalo milk	3.1×10^{8} CFU/mL	13 weeks	body weight and biochemical parameters no difference<br>serum IL-6 level ↓	not found	not found	not found	not found	not found
Feng et al., 2022 [114]	C57B-L/6 mice	male	6 weeks old	*Lactobacill-us rhamnosus* TR08	isolated from human gut	1×10^{8} CFU / animal / day	8 weeks	body weight and biochemical parameters no difference	not found	not found	not found	*Bifidobacterium* and *Bacteroides*↑<br>*Enterococcus*↓	acetic acid, propionic acid, and butyric acid↑
Chen et al., 2022 [115]	C57B L/6 mice	male	7 weeks old	*Lactobacill-us rhamnosus* strain LRH05	/	1×10^{9} CFU / animal / day	10 weeks	body weight↓<br>serum TG levels↓	TG levels↓<br>PGC-1α, NRF-1, SOD mRNA expression levels ↑	epididymal WAT and inguinal WAT weight↓<br>cross-sectional area of adipocytes in epididymal WAT, retroperitoneal WAT, and inguinal WAT↓<br>MOGAT1, ACOX1, IGF-1, MCP-1 mRNA expression levels↓	not found	Enterobacteriaceae, Ruminococcaceae, *Romboutsia*, *Lachnoclostridium*, *Eubacterium_coprostanoligenes_group*, *Lactobacillus rhamnosus*↑<br>*Ruminococcaceae UCG 014* and *Lactococcus lactis*↓	propionic, and butyric acids↑
Ivanovic et al., 2015 [116]	C57B-L/6 mice	male	6-8 weeks old	*Lactobacill-us rhamnosus* LA68<br>*Lactiplanti-bacillus plantarum* WCFS	Institute of Virology, Vaccines and Sera, “Torlak”, Serbia	2×10^{9} CFU	16 weeks	body weight↓<br>*Lactobacillus rhamnosus* LA68 group serum TC, HDL, Adiponectin levels ↓<br>*Lactiplantibacillus plantarum* WCFS group serum TG, LDL, Leptin levels↓	not found	not found	not found	not found	not found
Cheng and Liu, 2020 [117]	Balb/C mice	male	7 weeks old	*Lactobacill-us rhamnosus* GG	obtained from the American Type Culture Collection (ATCC; Manassas, VA, USA)	1×10^{8} CFU / animal / day<br>1×10^{10} CFU / animal / day	10 weeks	body weight no significant difference	lipid accumulation↓<br>lipid droplets↓	not found	the ratio of villus height to crypt depth↑	Firmicutes↑<br>Proteobacteria↓	not found
Sun et al., 2020	C57B-L/6 J mice	female	4 weeks old	*Lactobacill-us rhamnosus*	supplied by Wecake-b	1×10^{9} CFU / animal /	8 weeks	body weight↓<br>serum insulin, TG, TC, and	lipid accumulation↓	epididymal adipose tissues overall size of	not found	Firmicutes, Actinobacteria, *Faecalibaculum*,	not found

[118]				LRa05	io Co., Ltd. (Suzhou, China)	day		LDL-C levels↓		the adipocytes↓		*Desulfovibrio*, *Bifidobacterium*, *Streptococcus*, *Enterorhabdus*, and *Dubosiella*↓<br>Proteobacteria, Bacteroidetes, *Bacteroides*, *Alloprevotella*, *Intestinimonas*, and *Oscillibacter*↑	
Fang et al., 2019 [119]	ApoE-/- mice C57BL/6J mice	male	8 weeks old	*Lactobacillus rhamnosus* GR-1	not found	5 × 10^7 CFU / animal / day<br>5 × 10^8 CFU / animal / day	12 weeks	body weight↓<br>serum ALT, AST levels ↓<br>high dosage oxLDL, MDA, TNF-α, MCP-1, IL-6 levels ↓	not found	not found	not found	not found	not found
Ukibe et al., 2015 [120]	C57BL/6 J mice	male	6 weeks old	*Lactobacillus gasseri* SBT2055	isolated from human faeces	1 × 10^11 CFU/g	6 weeks	body weight no significant difference<br>fasting glucose levels↓	not found	abdominal fat weight no significant difference<br>CCL2, CCR2 and LEP mRNA expression levels↓<br>the population of macrophages in adipose↓	not found	not found	not found
Kim et al., 2025 [121]	C57BL/6 J mice	male	8 weeks old	*Lactobacillus paragasseri* SBT2055	provided by MEGMILK SNOW BRAND Co., Ltd. (Tokyo, Japan) and supplied by Ju Yeong Ns Co., Ltd (Seoul, South Korea)	1×10^8 CFU / animal / day<br>1×10^10 CFU / animal / day	12 weeks	body weight↓<br>plasma TC, FFA, LDL-c, Leptin levels↓, HDL-c, Adiponectin levels ↑	not found	Epididymal, Perirenal, Mesenteric, Retroperitoneum, Visceral, Subcutaneous white adipose tissues↓<br>epididymal WAT Adipocyte number↑<br>epididymal WAT PGC1α, CPT1a protein levels↑	FABP1, FABP2, FATP4, CD36, APOB48 mRNA expression levels↓, ABCG8 levels↑	Bacteroidota↑<br>Firmicutes/Bacteroidota ratio↓	not found
Lee et al., 2021 [122]	C57BL/6 J mice	male	10 weeks old	*Lactobacillus johnsonni* 3121<br>*Lactobacillus*	not found	1 × 10^10 CFU / animal / day	12 weeks	body weight↓<br>serum TC, LDL and VLDL levels↓	TC levels↓<br>lipid droplet↓	epididymal and inguinal adipose tissue weight↓<br>size of the adipocytes↓	not found	Firmicutes/Bacteroidota ratio ↓<br>*Roseburia* spp↓<br>*Faecalibacterium prausnitzii* and	not found

				rhamnosus 86<br>*Pediococcus pentosaceus* KID7						*L. johnsonni* 3121 and *L. rhamnosus* 86<br>PPARγ, C/EBPα, LPL, CD36 mRNA expression levels↓<br>*L. johnsonni* 3121 aP2, FASN and ACC mRNA expression levels ↓		*Akkermansia muciniphila*↑	
Hong et al., 2023 [123]	C57B-L/6 mice	male	7 weeks old	*Lactobacillus johnsonii* JNU3402	isolated from the feces of a healthy infant	1 × 10^8 CFU/mL	14 weeks	body weight↓	liver weight↓<br>lipid accumulation↓<br>FFA, TG, AST, ALT levels↓<br>SREBP-1c, FAS, ACC mRNA expression levels↓	epididymal and inguinal adipose tissue weight↓	not found	not found	not found
Lee et al., 2024 [124]	C57B-L/6 mice	male	6 weeks old	*Lactobacillus curvatus* HY7601 *and Lactiplantibacillus plantarum* KY1032 (1:1 mix)	isolated from kimchi	1 × 10^8 CFU / animal / day<br>1 × 10^9 CFU / animal / day	7 weeks	body weight↓<br>serum adiponectin, HDL-c levels ↑<br>serum TG、TC, LDL-c levels↓	PPARα, Hmgcr, SREBP2, ABCG5, ABCG8, LXRα, LXR, and CYP7A1 mRNA expression levels ↑	epididymal and inguinal adipose tissue weight↓<br>adipocytes size↓<br>UCP1, SIRT1, PGC1α protein levels↑	ABCG5, LXRα mRNA expression levels↑	not found	not found
Park et al., 2013 [125]	C57B-L/6J mice	male	4 weeks old	*Lactobacillus curvatus* HY7601<br>*Lactiplantibacillus plantarum* KY1032	isolated from Korean traditional fermented cabbage	5 × 10^9 CFU / animal / day	18 weeks	body weight↓<br>plasma ALT, TC, leptin and insulin levels↓	PGC1α , CPT1 , CPT2, HSL, CYP7A1, LDLR and ACOX1 mRNA expression levels↑, LPL, FAS levels ↓	total white fat mass↓<br>the size of the adipocytes↓<br>epididymal fat FAS and SCD1, TNFα , IL6, IL1β, MCP1, UCP2 and LPL mRNA expression levels ↓ , HSL levels ↑	not found	*Bifidobacterium pseudolongum*↑	not found
Li et al., 2019 [126]	C57B-L/6 mice	male	4 weeks old	*Lactobacillus reuteri* FN041	isolated from human breast milk	1 × 10^9 CFU/mL	7 weeks	body weight↓<br>serum TG, TC, LDL-c, TNF-α levels ↓<br>serum HDL-c levels ↑	fat infiltration↓<br>FAS, SREBP-1c mRNA expression levels↓	testicular fat↓	occludin, ZO-1, claudin-6, pIgR mRNA expression levels↑	*Christensenellaceae R7 group*, *Bifidobacterium*, *Mucispirillum*, *Acetatifactor*, *Acetivibrio ethanolgignens group*, *Peptococcus*, *Eisenbergiella*, and	propionic acid, and butyrate↑

												Clostridium sensu stricto 1. Clusters III and IV↑ *Ruminiclostridium 5*, *Turicibacter*, *Enterorhabdus*, the *Eubacterium coprostanoligenes group*, and *Coriobacteriaceae UCG 002*↓	
Abot et al., 2024 [127]	C57B-L/6J mice	male	9 weeks old	*Limosilact-o bacillus reuteri* BIO7251	isolated from Corsican clementin e in HFD fed mice	1 × 10^9 CFU / animal / day	5 weeks	body weight↓ plasma FFA levels↑	not found	subcutaneous adipose tissue↓ adipocytes number↓ ACC1, F4/80, CD68 mRNA expression levels↓	not found	not found	not found
Zheng et al., 2021 [128]	C57B-L/6J mice	male	5 weeks old	*Lactobacill-us rhamnosus* FJSYC4-1 *Lactobacill-us reuteri* FGSZY33-L6	isolated from the feces of healthy subjects	5 × 10^9 CFU/mL	12 weeks	body weight ↓ serum TC, LDL-c, fasting blood glucos levels↓	pathology score↓ IL-6 levels↓	adipose weight↓	not found	*L. reuteri* FGSZY33L6: *Romboutsia* and Desulfovibrioceae↑ *L. rhamnosus* FJSYC4-1: *Ruminiclostridium*, *Lachnospiraceae_U-CG_006* and *Lactobacillus*↑	butyric acid↑
Park et al., 2019 [129]	C57B-L/6 mice	male	7 weeks old	*Lactobacill-us amylovorus* KU4	not found	1.0 × 10^8 CFU/mL	14 weeks	body weight↓ plasma glucose levels and fasting insulin levels↓	TG levels↓	liver, epididymal, and inguinal WAT weight↓ adipocyte size in eWAT and lipid droplet↓ TG levels↓ UCP1, PPARγ, and PGC-1α proteins levels↑	not found	not found	not found
Liang et al., 2021 [130]	C57B-L/6J mice	male	3 weeks old	*Ligilactoba-cillus Salivarius* LCK11	Dairy Science and Probiotic Engineeri ng Lab, Harbin Institute of Technolo-gy	5 × 10^8 CFU/mL	8 weeks	body weight ↓ serum TG, CHOL, ALT and AST levels↓	TG and TCHO levels↓	including epididymal white adipose tissue, perirenal white adipose tissue, and inguinal white adipose tissue weights↓	PYY, TLR2 and NF-κB mRNA and protein levels↑	Bacteroidetes, Bacteroidales, Coribacteriales, and Verrucomicrobiales↑ Clostridiales, Deferribacterales, and Desulfovibrionales↓ Firmicutes/Bacteroide tes ratio↓	not found

					(Harbin, China)							
Kim et al., 2017 [131]	C57B-L/6 mice	male	4 weeks old	*Lactobacill-us kefiri* DH5	Isolation of lactic acid bacteria from kefir	2 × 10^8 CFU	6 weeks	body weight ↓<br>plasma TG and LDL-c levels↓	liver weight↓<br>lipid droplets↓	epididymal adipose tissue↓<br>PPAR-α, FABP4, and CPT1 mRNA expression levels ↑	not found	Proteobacteria and Enterobacteriaceae↓	not found
Zeng et al., 2020 [132]	C57B-L/6J mice	female	15-16 weeks old	*Lactobacill-us pentosus* S- PT84	provided from Suntory Wellness Ltd. (Kyoto, Japan)	0.06% (w/w)<br>0.6% (w/ w)	20 weeks	plasma endotoxin, LBP, MCP-1, TNF-α, TG, LDL-c, ALT insulin levels↓, adiponectin, HDL-c levels ↑	not found	adiponectin and IL-10 levels↑<br>macrophage infiltration in white adipose tissues↓<br>PPAR-γ, IRS-1 protein levels ↑	ZO-1, JAMA, claudin1, claudin 3, claudin 4, claudin 5, and claudin 7 mRNA expression levels↑	not found	not found
Jang et al., 2024 [133]	C57B-L/6J mice	male	4 weeks old	*Lactobacill-us delbrueckii subsp. lactis* CKDB001	not found	1 ×10^9 CFU/g	24 weeks	serum insulin, TG, ALT levels↓	liver weight↓<br>TG levels↓<br>p-AMPK protein levels↑<br>SREBP-1c mRNA expression levels↓	Epididymal adipose tissue leptin mRNA expression levels↓	not found	not found	not found
Song et al., 2023 [134]	Balb/c mice	male	5-7 weeks old	*Lactobacill-us coryniform-is* subsp. *torquens* T3	screened from traditional yaks' milk cheese from Lhasa	1 ×10^9 CFU/mL	6 weeks	body weight↓<br>serum TG, TC, IL-6, TNF-α, LPS levels↓	lipid droplets↓<br>TC, TG, AST, ALT levels↓<br>NF-κB, TNF-α, and IL-1β mRNA expression levels↓, NQO-1 and SOD levels ↑<br>KEAP1 protein levels↓	white adipose tissue weight↓<br>adipocyte deformation and inflammation↓<br>UCP1 protein levels↑	ZO-1, Claudin-1, and Occludin mRNA expression levels↑	*Harryflintia*, *Intestinimonas*, *Helicobacter*, *Angelakisella*, *Mucispirillum*, and *Ruthenibacterium*↓<br>*Streptococcus* and *Alistipes*↑	propionic acid, isobutyric acid, and valeric acid↑<br>hexanoic acid↓

Supplementary table S2 Effects of oral administration of a strain (belonging to the *Bifidobacterium*) on mice fed a high-fat diet

Refer-ences	Speci-es	Sex	Age or weight	Probiotic/Po-tential Probiotic	Resouces	Bacterial concentra-tion	Trea-tment cycle	Body weight and serum biochemical indicators	Liver	Adipose tissue	Intestinal	Gut microbe	SCFA and BA content
Rahman et al., 2021 [135]	C57B-L/6 mice	male	6 weeks old	*Bifidobacter-ium longum* subsp. *infantis* YB0411	isolated from infant stools	1 × 10^9 cells/kg	7 weeks	body weight ↓	not found	visceral and epididymis white adipose tissues lipid droplet area↓	not found	not found	not found
Kou et al., 2023 [136]	C57B-L/6J mice	male	4-6 weeks old	*Bifidobacter-ium longum* subsp. *infantis* FB3-14	screened from the feces of breastfed healthy infants	1 × 10^9 CFU / animal / day	8 weeks	body weight ↓ serum TC and LDL-C levels↓	liver weight↓ serum LPS, IL-6, IL-1β, TNF-α levels ↓	epididymal fat weight↓ adipocyte size↓ IL-6 and TNF-α mRNA expression levels↓	ADIPOR1, ADIPORQ mRNA expression levels↑	Firmicutes/Bacteroides ratio↓ Verrucomicrobiota, unclassified_Muribaculaceae, *Akkermansia muciniphila*, *Bifidobacterium*, *Lachnospiraceae_NK4A136_group*, *unclassified_Oscillospiraceae*↑	butyric acid↑
In et al., 2019 [137]	C57B-L/6 mice	male	6 weeks old	*Lactobacillus plantarum* LC27 *Bifidobacterium longum* LC67	not found	1 × 10^9 CFU / animal / day	8 weeks	body weight ↓ blood TG, TC, LDL-c, TNF-α, LPS, ALT, AST levels↓, HDL-c levels ↑	liver weight ↓ TG, TC, TNF-α, LPS, MPO levels ↓ iNOS, p-p65, p-Iκ-Bα, α-SMA protein levels ↓ p-AMPK, Claudin-1, Occludin protein levels ↑	not found	p-AMPK, claudin-1 and occludin protein levels↑ iNOS, p-p65, p-Iκ-Bα, α-SMA protein levels↓	Firmicutes/Bacteroidetes ratio↓ Firmicutes, Bacteroidetes, Proteobacteria, Defferibacteres↓	not found
Wu et al., 2020 [138]	C57B-L/6J mice	male	13 ± 2 g	*Bifidobacter-ium longum* subsp. *longum* BL21	supplied by Wecake-bio Co., Ltd. (Suzhou, China)	1 × 10^9 CFU / animal / day	8 weeks	body weight↓ serum TG, TC, LDL-c levels ↓	lipid accumulation↓	the size of the adipocytes↓	not found	Firmicutes/Bacteroidetes ratio↓ *Lachnoclostridium*, *Alistipes*, *Akkermansia*, *Bacteroides*, *Roseburia*, *Alloprevotella*, *Oscillibacter*, and unidentified_Lachnospiraceae↑ *Lactobacillus*, *Faecalibaculum*, *Desulfovibrio*, and unidentified_Ruminococcaceae↓	not found
Lim et al., 2017 [139]	C57B-L/6 mice	male	6 weeks old	*Bifidobacter-ium adolescentis* IM38	not found	2 × 10^9 CFU / animal / day	6 weeks	body weight↓ blood LPS levels ↓	not found	epididymal fat weight↓	ZO-1, occludin, and claudin-1 protein levels↑ MAPKs ERK, JNK, and p38, TNF, IL-β	Proteobacteria/Bacteroidetes ratio↓	not found

											protein levels↓		
Wang et al., 2020 [140]	C57B-L/6J mice	male	4 weeks old	*Bifidobacter-ium adolescentis* (FSDJN4-N3, HuNan2016 7-2, CCFM8630)<br>*Lactobacillus rhamnosus* (LGG, FHeNJZ7-1, FJSWX10-1)	isolated from human faeces in China	1 × 10^9 CFU/mL	23 weeks	body weight ↓<br>serum TG, TC, LDL-c, ALT, AST changes in different group	TG, TC, LDL-c, TNF-α, IL-1β, IL-6 changes in different group	not found	ZO-1, occludin, and claudin-1 mRNA expression levels changes in different groups	Firmicutes/Bacteroidetes ratio↓<br>*Clostridium* and *Streptococcus*↓<br>*Bifidobacterium* ↑	Acetic acid, Propionic acid, Butyric acid, Isobutyric acid changes in different groups
Ma et al., 2022 [141]	C57B-L6J mice	male	7 weeks old	*Bifidobacter-ium animalis* subsp. *lactis* LKM512	purchased from Meito Sangyo Co., Ltd. (Tokyo, Japan)	1 × 10^8 CFU<br>1 × 10^9 CFU	12 weeks	plasma TC, TG, NEFA, LPS levels↓<br>fasting glucose and insulin levels↓	liver weight↓<br>lipid droplet accumulation↓<br>ACC, SCD1, PKM2, G6P, F4/80, MCP1, CCL5, IL-6, iNOS mRNA expression levels↓, CPT1 levels↑	IL-6, iNOS mRNA expression levels↓	colon length↑<br>ZO-1, Claudin1, Occludin protein levels↑<br>MUC1, MUC2, DEFA, REG3G mRNA expression levels↑<br>F4/80, MCP1, CCL5, CD11C, CD3, CD4, CD8 mRNA expression levels↓	Proteobacteria, *Alistipes*, *Rikenellaceae RC9 gut group*, *Rikenella, Ruminococcaceae NK4A214 group*, *Romboutsia, Peptococcus*↓<br>*Anaerotruncus*, *Mucispirillum*, *Lachnospiraceae NK4A136 group*, *Enterorhabdus*, *Blautia*, *Acetatifactor*, *Butyricicoccus*, *Faecalibaculum*↑	not found
Zhang et al., 2024 [142]	C57B-L/6J mice	male	5 weeks old	*Bifidobacter-ium animalis* subsp. *lactis* MN-Gup	not found	2 × 10^9 CFU/kg<br>1 ×10^10 CFU/kg	13 weeks	not found	not found	not found	epithelial cell damage and sloughing↓	*Bifidobacterium*↑<br>*Escherichia-Shigella* and *Staphylococcus*↓	acetate↑
Ban et al., 2023 [143]	C57B-L/6J mice	male	6 weeks old	*Bifidobacter-ium lactis* IDCC 4301	Breast-fed infant's feces	1 × 10^8 CFU/day<br>5 × 10^8 CFU/day	12 weeks	body weight ↓<br>serum TC, LDL-c, leptin levels ↓	AST, ALT levels↓	adipose tissue weight ↓<br>adipocyte sizes↓<br>epididymal adipose tissue FAS, PPARγ, C/EBPα mRNA expression levels↓, HSL, PPARα levels↑	not found	not found	not found

Kondo et al., 2010 [144]	C57BL/6J mice	male	5 weeks old	*Bifidobacterium breve* strain B-3	isolated from the feces of infants	1 × 10^8 CFU/day 1 × 10^9 CFU/day	8 weeks	body weight ↓ serum TC, Glucose, Insulin levels↓	not found	epididymal adipose tissue weight↓ epididymal fat pad Adiponectin mRNA expression levels↑	Proglucagon mRNA expression levels↑	*Bifidobacteria*↑	not found
Cano et al., 2013 [145]	C57BL-6 mice	male	6-8 weeks	*Bifidobacterium pseudocatenulatum* CECT 7765	Spanish Type Culture Collection	5 ×10^8 CFU	7 weeks	body weight ↓ serum TC, TG, glucose, leptin, IL-6, MCP-1, IL-10 levels↓, IL-4 level ↑ fasting insulin level ↓	steatosis↓	adipocyte size no significant difference	the number of fat micelles in the enterocytes↓	*Bifidobacterium* spp.↑ Enterobacteriaceae↓	not found
Rahman et al., 2023 [146]	C57BL/6 mice	male	6 weeks old	*Bifidobacterium bifidum* DS0908 *Bifidobacterium longum* DS0950	isolated from human fecal	1 × 10^9 cells/kg	7 weeks	body weight ↓ serum TG, CHO, LDL levels↓	not found	adipocyte size↓	not found	not found	not found

Supplementary table S3 Effects of oral administration of a strain (other probiotics / potential probiotic) on mice fed a high-fat diet

References	Species	Sex	Age or weight	Probiotic/Potential Probiotic	Resouces	Bacterial concentration	Treatment cycle	Body weight and serum biochemical indicators	Liver	Adipose tissue	Intestinal	Gut microbe	SCFA and BA content
Huang et al., 2023 [147]	C57BL/6J mice	male	5 weeks old	*Bacillus coagulans* BC69	isolated from thick broad bean sauce	3×10^5 CFU	16 weeks	body weight↓ serum TNF-α level↓	liver weight↓ inflammatory cell infiltration↓	not found	not found	*Bifidobacterium*↑ *Ileibacterium*, *Dubosiella*↓	acetate and butyrate↑
Kim et al., 2018 [148]	C57BL/6J mice	male	5 weeks old	*Bacillus sonorensis* JJY12-3 *Bacillus paralicheniformis* JJY12-8 *Bacillus sonorensis* JJY13-1 *Bacillus sonorensis* JJY 13–3 *Bacillus sonorensis* JJY 13–8	isolated from long-term fermented soybean paste	1 x 10^8 CFU/day	13 weeks	body weight↓	liver weight↓ TNFα, INFγ , MCP-1, IL-12 mRNA expression levels↓ fat deposition↓ PGC1α protein level↑	subcutaneous adipose tissue, mesenteric adipose tissue, interscapular adipose tissue↓ TNFα, INFγ, MCP-1, IL-6 CD36, LDLR, SREBP1c, ACC, FAS, SCD1 mRNA expression levels↓	tight junction-associated proteins, Occludin, ZO-1 mRNA expression levels↑	*Bacteroides*↓	acetate↓
Wang et al., 2019 [149]	C57BL/6J mice	male	6 weeks old	*Bacillus amyloliquefaciens* SC06	stored in China Center for Type Culture Collection (No. M 2012280)	1 x 10^8 CFU/g	8 weeks	serum leptin, IL-6, TNF-α levels↓	steatosis↓ CAT level↑, MDA level↓	subcutaneous fat weight ↓ size of adipocytes↓	not found	Firmicutes/Bacteroidetes ratio↓ *Prevotella*↓	not found
Hashemnia et al., 2023 [150]	C57BL/6J mice	male	6 weeks old	*Bacillus coagulans* T4	IBRC-N10791	1 x 10^9 CFU / animal / day	18 weeks	body weight↓ fasting glucose levels↓ serum TG, insulin, Leptin, Resistin levels↓	not found	visceral fat and subcutaneous fat weight↓ SAT Adgre1, IL-1β, IL-6, Adipoq, TLR2, TLR4, SREBP1, FASN, CPT1A mRNA expression levels↓, Mrc1, Arg1, Itgax, Nos2, IL-10 levels↑ EAT Adgre1, Nos2, Itgax, TNF, IL-1β, IL-6, CCL2, Adipoq, TLR4 mRNA expression levels↓, Mrc1, Arg1, IL-10, SREBP1, FASN, ACACA, CPT1A levels↑ white adipose tissue	not found	Firmicutes/Bacteroidetes ratio↓ *Lactobacillus* and *Faecalibacterium*↑	Propionate and acetate↑

Cao et al., 2019 [151]	C57B-L/6J mice	male	8 weeks old	*Bacillus licheniformis*	obtained from the SLAC Laboratory Animal Central (Changsha, China)	1 x 10^8 CFU / animal / day	8 weeks	body weight↓ serum TG levels↓	TG levels↓	Epididymal fat weight↓	not found	*Bilophila*, *Bacillus*, *Enterorhabdus*, *unidentified_Rumi-nococcaceae*,and *Marvinbryantia*↑	not found
Wen et al., 2024 [152]	C57B-L/6J mice	male	8 weeks old	*Bacteroides vulgatus*	not found	6-7 ×10^8 CFU/mL	12 weeks	body weight↓ serum TC, TG, HDL-c, LDL-c levels↓	liver weight↓	epididymal adipocyte size ↓	chylomicron uptake↓	not found	not found
Li et al., 2024 [153]	C57B-L/6J mice	male	25.0-27.0 g	*Bacteroides thetaiotaomicron* ATCC 29,148	derived from ATCC	1 x 10^8 CFU / animal / day	12 weeks	body weight↓ serum TG, CHO levels↓ non-fasting blood glucose and fasting insulin levels↓	liver index↓ TG, CHO levels↓ palmitoleic/7-hexadecenoic acid (C16:1) levels↓	not found	not found	Firmicutes/Bactero-idetes ratio↓	not found
Sun et al., 2024 [154]	C57B-L/6J mice	male	4-5 weeks old	*Bacteroides ovatus*	isolated from 20 healthy Chinese people	2 x 10^9 CFU	12 weeks	body weight↓ serum LPS, CD163, IL-1β, TNF-α levels↓	liver weight↓ hepatic steatohepatitis↓ PPARα mRNA expression level↑, FASN, SCD1, ACACA, SREBF1 levels↓	not found	not found	Firmicutes/Bactero-idetes ratio↓ *Ruminococcus_tor-ques_group*, *Ruminococcus_ga-uvreauii_group*, *Erysipelatoclostrid-ium*↓ *Lachnospiraceae NK4A136_group*, *norank_f__Oscillo-spiraceae*, *Colidextribacter*↑	not found
Wang et al., 2020 [155]	C57B-L/6N mice	male	9 weeks old	*Pediococcus pentosaceus* PP04	isolated from the Northeast pickled cabbage (China)	1 x 10^9 CFU / animal / day	8 weeks	body weight↓ serum TC, TG, LDL-c, FFA, Leptin, MDA levels↓, SOD, GSH-Px levels↑	TC, TG, SOD, GSH-Px levels↑ p-AMPK, p-ACC1, MTP, PPARα, CPT1, NRF2 protein levels↑ SREBP-1c, FAS, SCD1, CYP2E1 protein levels↓	epididymal fat index and perirenal fat index↓	not found	not found	not found

Wang et al., 2021 [156]	C57BL/6N mice	male	9 weeks old	*Pediococcus pentosaceus* PP04	isolated from the Northeast pickled cabbage (China)	1 x 10^9 CFU / animal / day	8 weeks	not found	not found	not found	Occludin, Claudin-1 and ZO-1, NRF2 protein levels↑<br>TLR4, MyD88, pIκB-α/IκB-α, pNF-κB/NF-κB, CYP2E1 protein levels↓<br>IL-6, IL-1β, CYP2E1 mRNA expression levels↓, HO-1, NQO1 level↑<br>CAT, SOD levels↑	Firmicutes/Bacteroidetes ratio↓<br>*Pediococcus*, *Coprococcus*, *Allobaculum*, *Faecalibacterium* and *Butyricicoccus*↑<br>*Bacteroides* and *Pseudomonas*↓	not found
Yang et al., 2022 [157]	C57BL/6 mice	male	6 weeks old	*Clostridium cochlearium*	isolated and obtained from the Department of Nutrition and Food Science Wayne State University	2 x 10^9 CFU / animal / day	17 weeks	body weight↓<br>fasting blood glucose, Insulin↓	not found	fat mass↓	not found	not found	Acetate, Propionate, Butyrate ↓
Luo et al., 2024 [158]	C57BL/6J mice	male	4 weeks old	*Clostridium tyrobutyricum* ATCC25755	Ohio State University	1 x 10^7 CFU / mL<br>1 x 10^8 CFU / mL	12 weeks	body weight↓<br>serum TG, LDL-c levels↓	liver weight↓<br>TC, TG, NEFA levels↓<br>PPARγ mRNA expression levels↓, AMPK, PPARα, HSL levels↑	not found	Villus height and the V/C, colon length↑<br>TNF-α, IL-1β, and IL-6 mRNA expression levels↓, IL-10, Occludin levels↑	*Deferribacteres*, *Blautia* and *Mucispirillum*↓<br>*Colidextribacter*, *Intestinimonas*, *Acetatifacter*, *Lachnospiraceae NK4A136_group*, *GCA-900066575*, and *Tuzzerella*↑	Propionic acid↑
Benvenuti et al., 2023 [159]	C57BL/6J mice	male	5 weeks old	*Enterococcus faecium* SF68	not found	1 x 10^8 CFU / animal / day	8 weeks	body weight↓<br>plasm IL-1β↓	not found	not found	acidic mucins level↑<br>tight junction protein, NF-κB level↑<br>TLR4 protein levels↓<br>SMCT1 protein levels↑	Firmicutes and Bacteroides↓	not found
Xu et al., 2023 [160]	C57BL/6J mice	male	8 weeks old	*Blautia producta*	Beijing QuantiHealth Technology Co., Ltd. (Beijing, China)	1 x 10^9 CFU / 0.2 mL	8 weeks	body weight↓<br>blood TG, TC, and LDL-c levels↓	liver TG and TC levels↓	lipid accumulation↓	not found	*Akkermansia*↑<br>*Desulfovibrio piger* and *Desulfovibrio* sp. G11↓	not found

Castro-Rodríguez et al., 2020 [161]	C57BL/6 mice	male	5-6 weeks old	*Leuconostoc mesenteroides subsp. mesenteroides* SD23	isolated from aguamiel of Agave salmiana	1 × 10^10 CFU/mL	14 weeks	body weight↓<br>serum Glucose, TC, Leptin levels↓	fat liver area↓<br>SREBP-1c, FAS, TLR4, CD14, TNF-α mRNA expression levels↓, IL-10 level↑	adipose tissue weight↓<br>adipocyte area↓	Villus height and width, crypts depth↓, goblet cells↑	not found	not found
Huang et al., 2025 [162]	C57BL/6 mice	male	5 weeks old	*Roseburia hominis* A2-183	obtained from the Leibniz Institute DSMZ-German Collection of Microorganisms and Cell Cultures	1 x 10^9 CFU	11 weeks	body weight↓<br>serum TC, TG levels↓	lipid accumulation ↓<br>TG, ALT, AST levels ↓	adipocyte diameter↓<br>white adipose tissue area↓<br>brown adipose tissue CIDEA mRNA expression levels↑	not found	*Faecalibacterium prausnitzii*, *Dysosmobacter welbionis*, *Parabacteroides goldsteinii*, and *Lactobacillus johnsonii*↑<br>*Olsenellauli*↓	not found
Lu et al., 2024 [163]	C57BL/6 mice	male	6-8 weeks old	*Coprococcus*	not found	1 x 10^9 CFU / animal / day	16 weeks	serum ALT, TC, LDL-c levels↓	liver lipid accumulation, inflammation, and fibrosis↓<br>TG level↓<br>FASN, SCD1, CD36, TNF-α, IFNY, TIMP1 mRNA expression levels↓	not found	not found	not found	not found
Wu et al., 2023 [164]	C57BL/6 mice	male	6 weeks old	*Akkermansia muciniphila MucT* (ATCC BAA-835)	purchased from ATCC	1.5 x 10^9 CFU	21 weeks	body weight↓<br>serum ALT, AST levels↓	liver weight↓<br>TG, insulin, leptin and resistin level↓<br>FGFR4 mRNA expression levels↑, CYP7A levels↓	subcutaneous, mesenteric, and epididymal white adipose tissue↓	Occludin, TJP1, FXR, SHP, FGF15 mRNA expression levels↑, TNF-α, F4/80 levels↓	*Alistipes*, *Lactobacilli*, *Tyzzerella*, *Butyricimonas* and *Blautia*↓<br>*Ruminiclostridium*, *Osclibacter*, *Allobaculum*, *Anaeroplasma* and *Rikenella*↑	total BAs↓<br>DCA and LCA levels↓
